# Understanding the impact of modiolus porosity on stimulation of spiral ganglion neurons by cochlear implants

**DOI:** 10.1038/s41598-024-59347-2

**Published:** 2024-04-26

**Authors:** Kiran K. Sriperumbudur, Revathi Appali, Anthony W. Gummer, Ursula van Rienen

**Affiliations:** 1 Institute of General Electrical Engineering, University of Rostock, Rostock, Germany; 2 Ageing of Individuals and Society, Interdisciplinary Faculty, University of Rostock, Rostock, Germany; 3 Life, Light and Matter, Interdisciplinary Faculty, University of Rostock, Rostock, Germany; 4 Department of Otolaryngology, University of Tübingen, Tübingen, Germany; 5 Department of Otolaryngology, University of Melbourne, Melbourne, Australia; 6 Research and Development, MED-EL Medical Electronics GmbH, Innsbruck, Austria

**Keywords:** Computational models, Neurodegeneration, Translational research

## Abstract

Moderate-to-profound sensorineural hearing loss in humans is treatable by electrically stimulating the auditory nerve (AN) with a cochlear implant (CI). In the cochlea, the modiolus presents a porous bony interface between the CI electrode and the AN. New bone growth caused by the presence of the CI electrode or neural degeneration inflicted by ageing or otological diseases might change the effective porosity of the modiolus and, thereby, alter its electrical material properties. Using a volume conductor description of the cochlea, with the aid of a ‘mapped conductivity’ method and an ad-hoc ‘regionally kinetic’ equation system, we show that even a slight variation in modiolus porosity or pore distribution can disproportionately affect AN stimulation. Hence, because of porosity changes, an inconsistent CI performance might occur if neural degeneration or new bone growth progress after implantation. Appropriate electrical material properties in accordance with modiolar morphology and pathology should be considered in patient-specific studies. The present first-of-its-kind in-silico study advocates for contextual experimental studies to further explore the utility of modiolus porous morphology in optimising the CI outcome.

## Introduction

**Figure 1:**
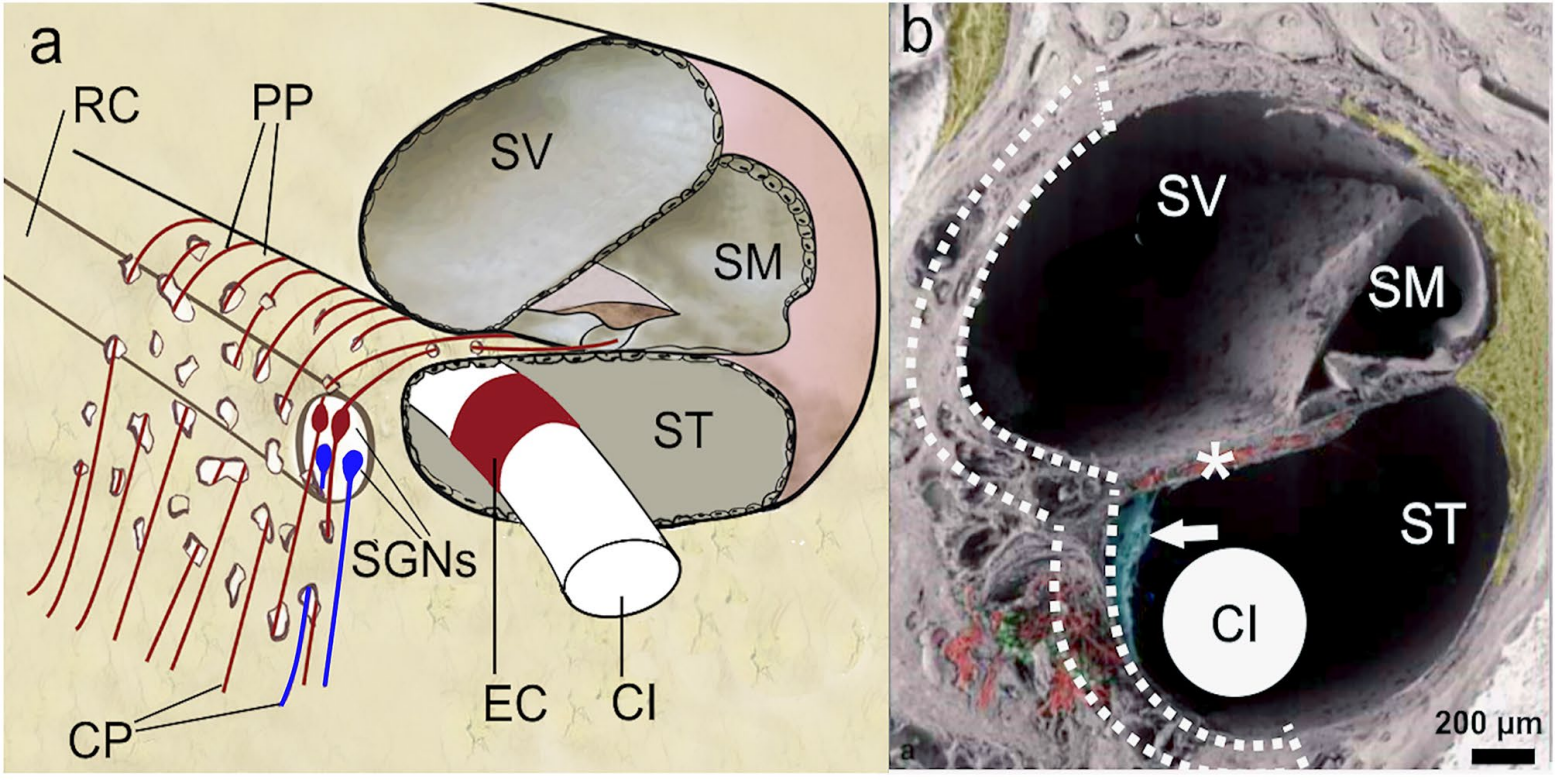
Location of the cochlear-implant electrode and the morphology of the porous modiolus. (**a**), Schematic cross-section of the cochlea, showing a cochlear implant (CI) inserted into one of the three fluid-filled canals, scala tympani (ST). The other two canals are called scala vestibuli (SV) and scala media (SM). The red rectangle, labelled EC, denotes one of the electrical contacts (or channels) comprising the CI. The CI stimulates the spiral ganglion neurons (SGNs), each of which, when intact, is comprised of a peripheral process (PP), a cell body called a spiral ganglion cell (SGC), and a central process (CP). The SGCs are located in a hollow, fluid-filled spiral canal called Rosenthal's canal (RC) that runs inside the porous bony modiolus, the cochlea axis. Two examples are shown of SGNs with intact peripheral processes (red SGCs), and two without (degenerated) peripheral processes (purple SGCs). Adapted with permission from Ref.^5^, their Fig. 2a. (**b**), Scanning electron microscopic (SEM) image of a cross-section of the lower basal turn of the human cochlea, with the schematic cross-section of a perimodiolar CI^30^ added into the image. Green: SGCs. Red: Auditory nerve fibres (also called peripheral axons or dendrites). Arrow: Mesothelial sheet guiding the dendrites through the osseous spiral lamina (*) to the SGCs. The dotted lines delineate the region used for modelling the electrical effects of modiolus porosity. Adapted with permission from Ref.^7^, their Fig. 18a.

**Figure 2:**
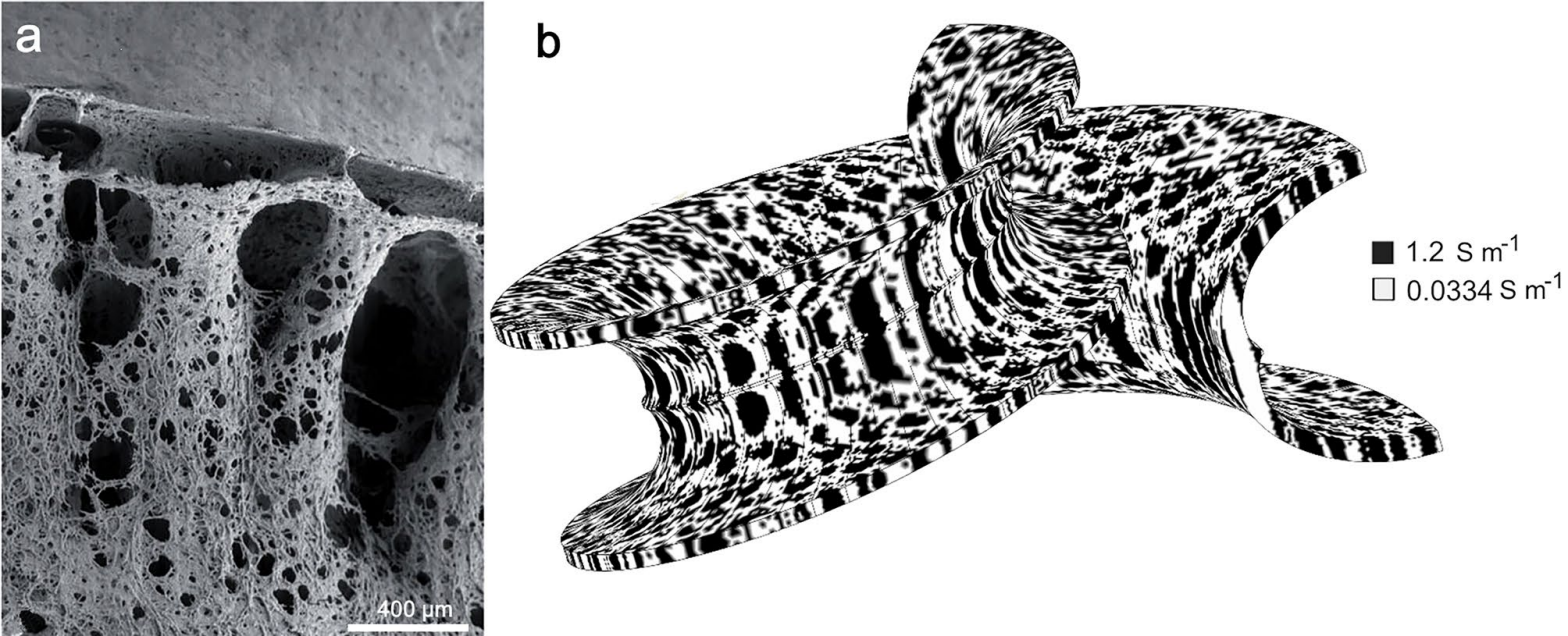
Image based porosity mapping. (**a**) Fenestrated bony columns of the modiolar bone in the lower basal turn of the human cochlea. Orientation is roughly transversal with scala tympani located at the lower aspect. With permission from Ref.^7^, their Fig. 18e. (**b**) Inhomogeneous electrical conductivity distribution on the computational subdomain of the porous modiolus modelled using the ‘mapped conductivity’ method (“Methods”). Briefly, the grey-scale intensities of the pixels of the SEM image in (**a**) were imported into the binary conditional Eq. (2), with ${\upalpha }$ = 0.5, to yield the conductivity at each pixel of the image, ignoring possible blockage of the porous boney complex. Black: pores (holes) with high conductivity equal to that of perilymph (1.2 S m^−1^). Pale grey: bone with low conductivity (0.0334 S m^−1^). Notice the similarity between the anatomical image in (**a**) and the conductivity map.

Primary cochlear neural degeneration induces sensory neural hearing loss, for threshold and suprathreshold stimuli^1–3^. In the pursuit of treating moderate-to-profound sensorineural hearing loss, a cochlear implant (CI) electrode array is surgically inserted into scala tympani (ST) to electrically stimulate the spiral ganglion neurons (SGNs) of the auditory nerve (AN) (Fig. 1a)^4–6^. The cell bodies of the SGNs, called the spiral ganglion cells (SGCs), are located in a hollow, fluid-filled spiral canal called Rosenthal's canal (RC) that runs spirally inside the bony modiolus, the cochlea axis (Fig. 1a)^7^. The modiolus is a thin, cone-shaped, multi-layered, inhomogeneous porous bony material (Figs. 1b, 2a)^7,8^. The porous nature of the modiolus suggests a rich fluid exchange between the perineural (and perivascular) spaces in the modiolus and the perilymphatic fluid of scalae tympani and vestibuli^9,10^.

Despite being celebrated for the one millionth cochlear implant^11^ and being the most successful neural prosthesis regarding both restoring neural function and the number of implantations^4^, a sizable number of CI users are considered to be ‘poor performers’, 10–50% dependent on the test criteria^12^. Even using the same CI device and stimulation parameters, implanted by similar surgical approaches and techniques, there is a formidable variability in the performance across CI users^12^. Many factors have been experimentally investigated to understand the basis of this so-called ‘enigma of poor performance’^12^. These include electrode design, speech coding and stimulation strategies, CI insertion depth, proximity to the modiolus, number of active electrodes, effective number of independent channels, temporal resolution, age at implantation, duration of deafness and the CI experience, residual hearing in both ears, number and health of surviving SGNs, patterns of neural degeneration, congenital sensory loss, tissue damage due to implantation, new bone or fibril formation around the electrode casing, ability of the brain to adapt and compensate for sensory loss, linguistic skills and neuro-cognition, and test–retest reliability of the speech tests^4,13–20^. Genetically determined SGC health accounts for about 18% of the variance of speech recognition outcomes for CI users^21^. The access resistance of the CI electrode increases with its proximity to the modiolus and the amount of bone^22^. Therefore, eventual intra-individual changes in the morphology of the modiolus might also contribute to the variance of the CI outcome. However, to date, there have been neither experimental nor modelling studies on the impact of the porous morphology of the modiolus on CI outcome.

It has been estimated that, for monopolar stimulation, a relatively small proportion of the current injected from a CI exits through the modiolus to excite the SGNs (16%^23^, 14%^24^), with the most significant part through the cochlear walls (54%^23^, 64%^24^) followed by the basal end of the cochlea (30%^23^, 22%^24^). Therefore, changes in the electrical conductivity of the modiolus are expected to have a significant and perhaps disproportionally large effect on the amount of current available to stimulate the SGNs.

Ageing or otological disease can inflict neural degeneration, modiolus ossification, or new bone growth^25^. In the case of neural degeneration, the volume fraction of neural (myelin) tissue in the modiolus would decrease. The resultant space created in the form of pores is presumably filled with perilymph. As a result, the macroscopic electric conductivity of the modiolus increases, since the electric conductivity of perilymph is higher than that of myelin tissue and bone. Likewise, the modiolus ossification or new bone growth would decrease the macroscopic electric conductivity of the modiolus. Such changes in the macroscopic electrical conductivity of the modiolus can influence its electrical responses to the applied electric field^26^. Micco et al.^27^, using the four-electrode reflection-coefficient technique in the in-vivo gerbil cochlea, showed that the electrical conductivity of the modiolus increased from 0.234 to 0.347 S m^−1^ when measured two months after the induction of neural degeneration by neomycin administration. Histology showing that Rosenthal’s canal was no longer filled with SGNs but with fibrous tissue and fluid explained the increased conductivity resulting from neural degeneration. The value of 0.234 S m^−1^ is up to three times larger than the conductivity found in^28,29^ for the in-vivo guinea-pig modiolus. According to Micco et al.^27^, the larger conductivity for gerbil is possibly due to the different cochlear bone densities of gerbil (thin and porous) and guinea pig (compact).

In several in-silico studies^23,31–34^, the electric conductivity of perilymph was assumed to be around 1.43 S m^−1^, similar to that of human cerebrospinal fluid (CSF) measured by Baumann et al.^35^. However, the proteome analysis conducted by Schmitt et al.^36^, on 34 CI candidates, shows human perilymph contains more perilymph-specific proteins than human CSF-specific proteins in the tested samples. According to^36^ the quantitative differences in protein content between perilymph and CSF are supposedly caused by cochlin, albumins, keratins, immunoglobulins, and apolipoproteins. How the concentration of these specific proteins influence the electric conductivity of perilymph is not yet known. However, a negative correlation between the protein concentration in human serum and its electric conductivity has been reported^37^. Hence, we assumed that the perilymph of CI candidates might have slightly lower electric conductivity due to the protein concentration difference caused by pathology induced perilymph-specific proteins compared to healthy perilymph or CSF. Consequently, we assigned 1.2 S m^−1^ to perilymph in the present study. This value is similar to the conductivity of artificial perilymph (1.25 S m^−1^) used for in-vivo intra-cochlear electrochemical impedance studies^38,39^.

The electrical conductivity of perilymph (1.2 S m^−1^) is much higher than that of myelin neural tissues (1.2 × 10^−6^ S m^−1^ per lamella^40^), so that with an increase of effective porosity, the macroscopic electrical conductivity of the modiolus increases accordingly. Conversely, an increase in the bone density due to modiolar ossification or new bone growth would impede the flow of perilymph, which would reduce the conductivity of the modiolus. Hence, it is a plausible conjecture that any change in the composition of the modiolus would impact its macroscopic electrical conductivity and, thereby, the CI performance. Therefore, it begs the question: How sensitive is the excitation of the AN by the CI to the morphology-dependent electrical properties of the modiolus?

In-vivo or in-vitro protocols to address such questions require a theoretical foundation before investing in animal or human experiments of different pathology. In-silico studies prove invaluable in such a scenario due to the scope of parameterizing electrical properties, the flexibility of modelling study-specific cochlear morphology, and the ease of visualizing electric field distributions on the cochlear interfaces^41–47^. To date, the only inclusion of porosity of the modiolar bone in studies of CI-elicited neural excitation patterns is one in which the microstructure of RC was resolved in order to include the trajectories of the nerve fibres in the finite-element model^45^. However, a suitable method for implementing the porous morphology of the modiolus in a volume conductor model of the cochlea is not available. The present study develops a mathematical model of the random distribution of the inhomogeneous porosity of the modiolar bone and incorporates it into a finite-element description of the electric field distribution induced by a CI.

Histological studies of human temporal bones suggest that the pore diameter along the modiolus, especially in the modiolar region of the osseous spiral lamina adjacent to RC, varies between 0.2 and 45 µm^9,10,48^. Inter-individual variations in the modiolus porosity reported in those studies imply that the modiolus conductivity would also vary accordingly. Moreover, variations in pore size and distribution imply an inhomogeneous electrical conductivity distribution throughout the modiolus along the cochlea. Although not attributed to the porosity of the modiolus specifically, conductivity variations in cochlear structures in each turn of the cochlea due to hydration, tissue density, and surface roughness were observed in animal experiments^49^. Thus, we suggest that in-silico investigations of the efficacy of the CI should map the inhomogeneous conductivity of the modiolus according to its porosity to capture the conductive nature of the porous modiolus realistically. However, to date, all contextual in-silico models have assumed the modiolus to be a non-porous bone with homogeneous conductivity.

In the present in-silico study, we introduce two methods to model the inhomogeneous distribution of the electrical conductivity of the porous modiolus in a volume conductor model of the human cochlea. The first method uses a scanning electron microscopic (SEM) image of the human modiolus to map the spatial distribution of electrical conductivity over the modiolus subdomain. The second method introduces an ad-hoc reaction–diffusion equation system to mathematically generate and manipulate the randomness of the inhomogeneous distribution of the pores while maintaining the volume occupied by the pores and, therefore, the overall porosity of the modiolus constant. By changing control parameters in the two methods, we simulate the effects of different degrees of neural degeneration and new bone growth on the electric fields in the modiolus and on the transmembrane potentials of SGCs in Rosenthal’s canal. We show that eventual changes in the porous morphology of the modiolus profoundly affect auditory-nerve stimulation. We conclude that assigning inhomogeneous conductivity to the modiolus in patient-specific in-silico studies is critically important for capturing the electrophysiological effects of neural degeneration and osteopathology on the efficacy of the CI.

## Results and discussion

We begin by examining the effect of modiolus porosity on electrical conductances, voltages, and fields as well as on the maximum transmembrane potentials of SGNs for three hypothetical morphological conditions of the modiolus bone (Case 1–3). The healthy modiolus is assumed to be composed of 40% bone, 40% myelin neural tissues and 20% perilymph-filled pores (“Methods”).

1. A homogeneous, very low conductive, non-porous modiolus with the isotropic, effective electrical conductivity of ${\upsigma }_{{\text{eff}}}=$ 0.0334 S m^−1^ (Eq. (4) with ${\upsigma }_{{\text{eff}}}\approx {\upsigma }_{{\text{U}}}$). This case quantitatively represents the worst-case scenario of filling the pores with non-conducting, extremely ossified bone or fibrous tissue^50^.
2. A morphologically realistic, inhomogeneous, porous modiolus having 50% porosity as a result of 75% neural degeneration. The mapped conductivity (${\upsigma }_{{\text{map}}}$) distribution for this modiolus is shown in Fig. 2b.
3. A homogeneous modiolus having 50% porosity with the isotropic, effective electrical conductivity calculated for the mapped conductivity distribution in Case2 using ${\upsigma }_{{\text{U}}}$ from Eq. (4) to yield ${\upsigma }_{{\text{eff}}}=$ 0.6334 S m^−1^.

**Figure 3:**
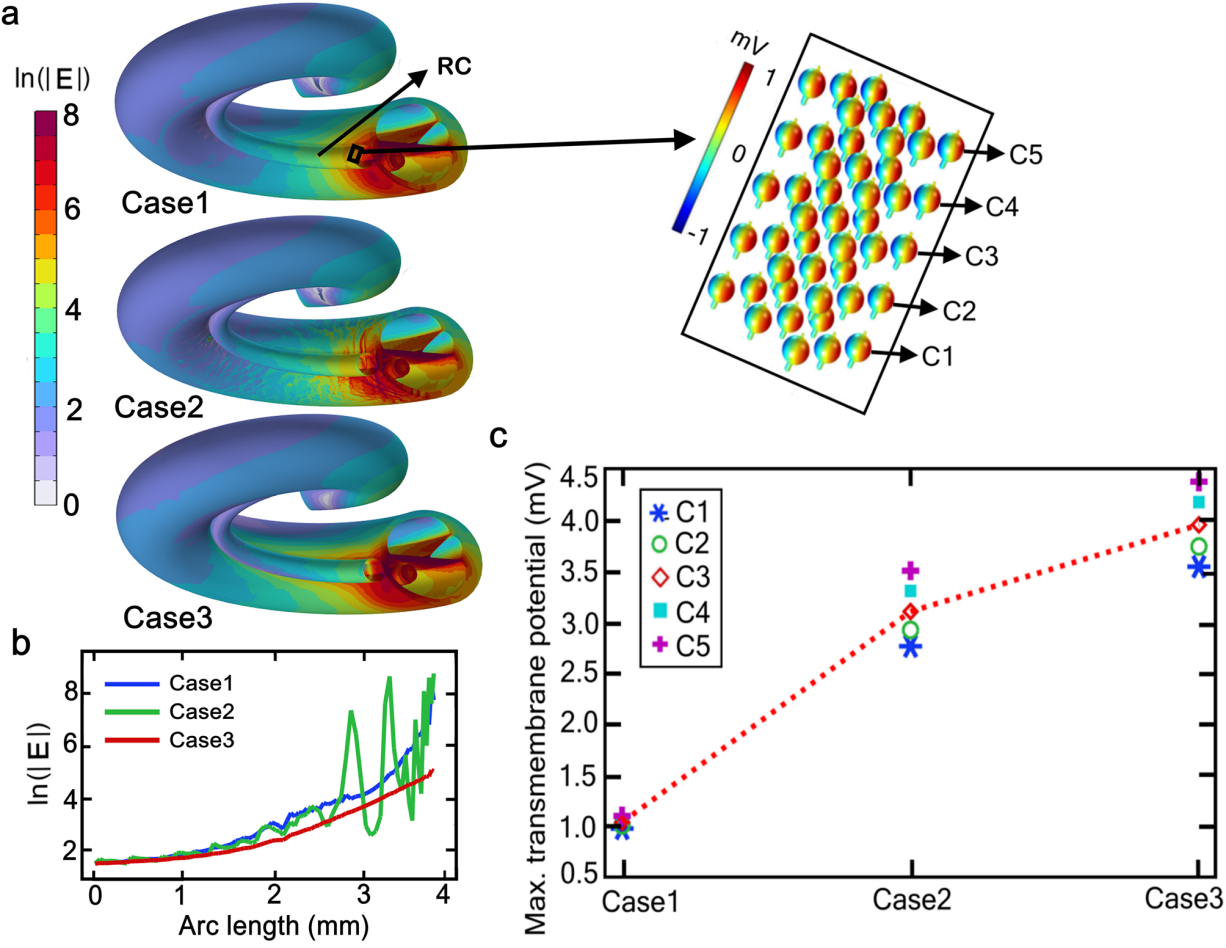
Electrical conditions in the cochlea in response to cochlear-implant stimulation for three hypothetical cases of modiolus porosity and conductivity. (**a**) Distribution of electric-field intensities in the modiolus. Case1: Non-porous modiolus, electrically isotropic of very low conductivity (0.0334 S m^−1^), Case2: Inhomogeneous porous modiolus (50% overall porosity), mapped (non-isotropic) conductivity (Fig. 2b), Case3: Homogeneous porous modiolus (50% overall porosity), effective (isotropic) conductivity 0.6334 S m^−1^. The stimulus voltage is 1 V DC. The electric field intensity magnitude is expressed in natural logarithms relative to 1 V m^−1^. Several subdomains are kept hidden for better visualization. Inset: Distributions of the transmembrane potentials over the surfaces of the spiral ganglion neurons (SGNs) for Case1. The position of the SGNs is delineated with a rectangular box in Rosenthal’s canal (RC) for Case1; they are located directly opposite the second electrode contact. Notice the fine structure in the electric field distribution for the case of inhomogeneous porosity (Case2). (**b**) Comparison of the electric field distribution pattern on the modiolus for the three cases as a function of a 4-mm arc length of the modiolus beginning from the basal-most region. c, Maximum transmembrane potential, V_max_, for the five SGNs (C1‒C5) highlighted with the inset. The red dotted line joining the V_max_ of C3 is drawn to aid visual comparison; it does not represent a functional relationship among the three cases. For the model, a generalized CI design with high-resistance silicon with 20 platinum electrode pads was implemented. The CI had a diameter of 0.6 mm and the electrode contacts had a length of 0.3 mm; their centres were separated by 0.9 mm and were located at a distance of 0.3 mm from the modiolus.

A comparison between the results for Case2 and Case3 is essential for deciding whether assigning a homogeneous effective conductivity to the modiolus is sufficient to reproduce the electrical response of the porous modiolus. A comparison between the results of Case1 and Case3 should yield a general idea about the impact of significant conductivity variations of the modiolus on AN stimulation. The results are presented in Fig. 3.

In Case1, due to the very low effective conductivity of the modiolus (0.0334 S m^−1^), a high electric field is confined to the vicinity of the active electrodes (Fig. 3a,b). And, for Case3, due to the higher effective conductivity (0.6334 S m^−1^) a very smooth electric field distribution can be seen on the modiolus, as also demonstrated by the smooth red curve in Fig. 3b. In contrast to Case1 and Case3, Case2 presents an inhomogeneous and random field distribution, as also demonstrated by the irregular green curve in Fig. 3b, due to the randomly mapped conductivities. The simulation clearly shows that the electric field distribution is profoundly affected by the morphology-based electrical conductivity of the modiolus.

A comparison of the maximal transmembrane potential (V_max_) induced on the indicated five SGNs (C1–C5) for the three cases is shown in Fig. 3c. Here, C5 is the closest and C1 is the farthest SGN from the modiolus in this first layer of the SGN matrix. Consequently, the induced potential is highest on C5 and lowest on C1. For the five SGNs, V_max_ is largest for the homogeneous porous modiolus of high conductivity (Case3) and smallest for the non-porous modiolus of very low conductivity (Case1). Indeed, the V_max_ in Case3 is almost four times larger than that in Case1 for a nearly 20-fold conductivity increase. This finding supports the suggestion of Malherbe and colleagues^51^ that the electrical conductivity of the skull and other bony interfaces around the CI electrode could profoundly affect the activation of the AN fibres.

Although the estimate of the effective conductivity of the modiolus in Case3 was based on the mapped conductivity from Case2, the V_max_ on the SGNs was larger by at least 25% (Fig. 3c). A possible reason for this apparent difference could be an overestimate of the effective conductivity by the Wiener upper bound (Eq. 4); that is, we assumed that the pores are orientated parallel to the applied electric field. If this assumption is essentially invalid, then the effective conductivity will be somewhere between the Wiener upper and lower bounds. Due to the spiral structure of the modiolus, it is not plausible to estimate the exact orientation of the pores relative to the electric field lines.

Additionally, the volume fraction of the bone-tissue network and pores in the modiolus could vary along the cochlea, resulting in different conductivity distributions in the basal, middle and apical regions of the cochlea, as reported in Kumar et al.^49^ for the gerbil. This possibility in humans can be ascertained by analysing the distribution and numbers of SGCs in both healthy and diseased cochleae; refer to Dhanasingh et al.^52^ for a detailed review. Therefore, it is expected that the morphology and curvature of the modiolus would influence the distribution of the electric potential within and along the spiral extent of RC. Thus, the differences in V_max_ for Case2 and Case3 (Fig. 3c) emphasize a weakness lurking in computational models which assign a single homogeneous conductivity to the entire modiolus.

The simulation results demonstrate that the implementation of modiolus porosity in in-silico cochlear studies could profoundly affect the distribution of electric potentials and fields in RC. Thus, utmost attention must be paid to the morphology of the modiolus when attempting patient-specific in-silico studies or clinical follow-up.

### Clinical relevance of intra- and inter-individual variations in the modiolus porosity

It is conceivable that otological disease may trigger intra-individual variations in the porous morphology of the modiolus, similar to Case1 or Case2. That means that for the exemplary volumes given in the preceding section, the effective conductivity of the healthy modiolus (0.2734 S m^−1^ before neural degeneration, as calculated in *Methods* using Eq. (4)) would drop to 0.0334 S m^−1^ if extreme neural degeneration were to fill the pores with non-conducting, extremely ossified bone or fibrous tissue as in Case1, or would rise to 0.6334 S m^−1^ due to 75% neural degeneration yielding 50% porosity as in Case2 (Supplementary material). Such changes are expected to impact profoundly on the efficacy of the CI over time post implantation. Considering reported anatomical variations of the cochlea^53,54^, inter-individual variations in the porosity specific to the respective anatomy can be expected. Therefore, the degree of neural or tissue degeneration and variations in the dimensions of the modiolus^55^ are supposed to influence the effective electrical properties of the modiolus. Although little is known from experimental or clinical studies, it is important to investigate how a broad spectrum of changes in the modiolus porosity inflicted by various otological diseases or electrode trauma might affect AN stimulation.

**Figure 4:**
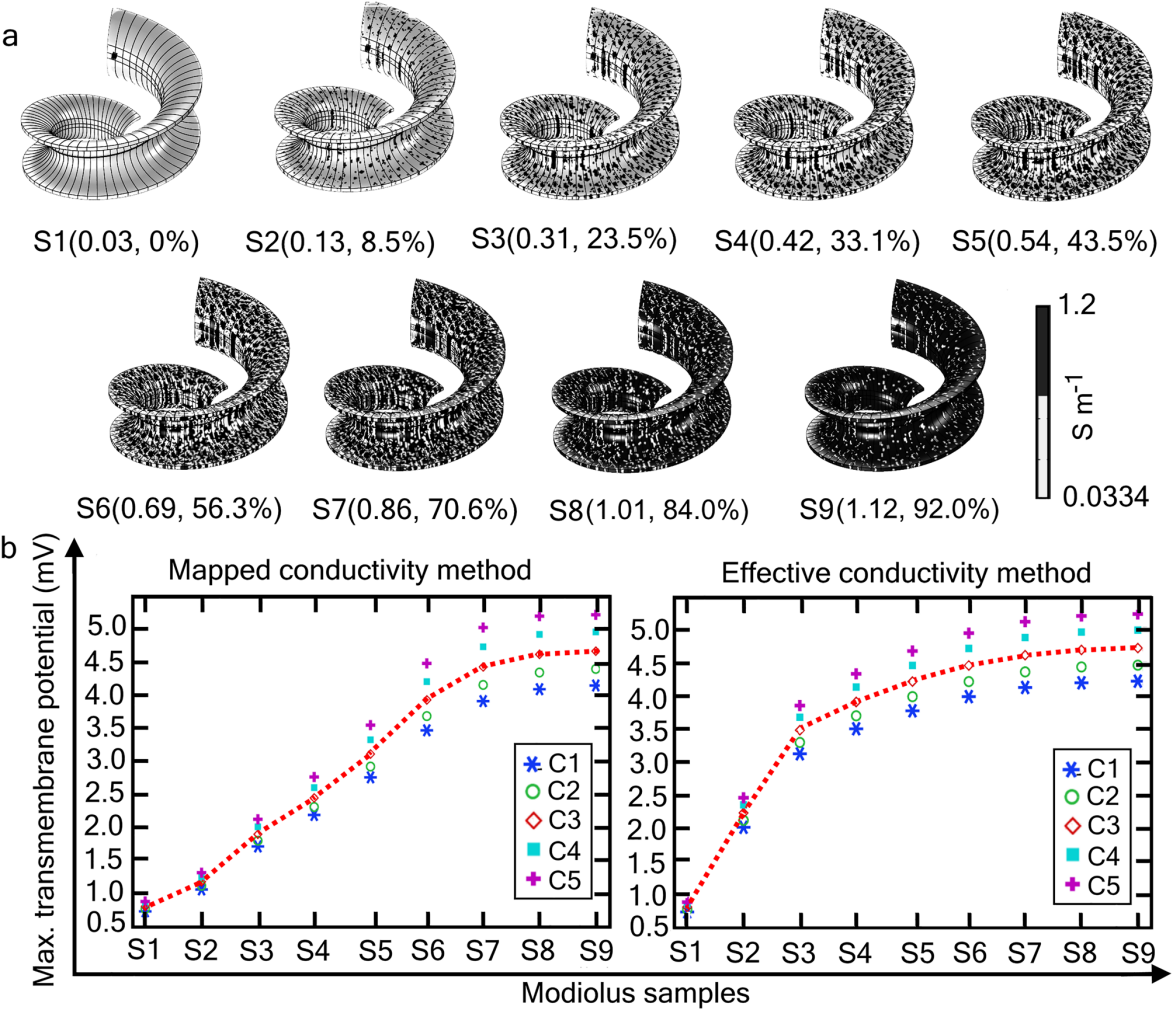
Maximum transmembrane potentials for modioli of different overall porosity. (**a**) Distribution of the mapped conductivity for nine hypothetical modiolus samples (S1‒S9) of different overall porosity and inhomogeneity (“Methods”). Each sample is designated by its estimated effective conductivity (S m^−1^) and overall porosity (%), written as S(effective conductivity in S m^−1^, overall porosity %). (**b**) Maximum transmembrane potential, V_max_, induced on the five SGNs (C1‒C5) shown in Fig. 3a (inset), for the nine modiolus samples, where the electrical conductivity for each modiolus sample was assigned using the *mapped* conductivity method (left panel), and the *effective* conductivity method (right panel). Red dotted line: Visual aid drawn through the C3 data points. Notice that for samples S2‒S5 (8.5 ≤ porosity ≤ 43.5%), V_max_ using the effective conductivity model is larger than that for the mapped conductivity model. Porosity and effective conductivity for the healthy modiolus are assumed to be 20% and 0.2734 S m^−1^, respectively.

To this end, we modelled nine samples of modiolus with *random* porosity by assigning 0, 0.15, 0.25, 0.35, 0.45, 0.65, 0.75, 0.85, and 0.95 to the intensity parameter (${\upalpha }$) in Eq. (2). Then, the effective conductivity of each sample was estimated using Eq. (4), with ${\upsigma }_{{\text{eff}}}\approx {\upsigma }_{{\text{U}}}$. Figure 4a shows nine samples (S1–S9) with their respective effective conductivities. Taking the proposed effective conductivity of the modiolus before osteopathy or neural degeneration as the reference for a healthy cochlea (0.2734 S m^−1^ for 20% porosity), the pathology of each sample is described below, in our own words, and designated symbolically as S (effective conductivity in S m^−1^, porosity %):

- S1(0.0334, 0%): worst-case scenario of modiolus ossification, severe occlusion of the pores with non-conducting, extremely ossified bone or fibrous tissue
- S2(0.1325, 8.5%): extreme modiolus ossification or fibrous tissue regrowth
- S3(0.3092, 23.5%): mild neural degeneration
- S4(0.4202, 33.1%): moderate neural degeneration
- S5(0.5410, 43.5%): high neural degeneration
- S6(0.6911, 56.3%): extreme neural degeneration with almost no neural tissue present in the modiolus
- S7(0.8575, 70.6%): moderate modiolus malformation
- S8(1.0136, 84.0%): extreme modiolus malformation
- S9(1.1149, 92.0%): modiolus not present

Image data are not available describing the eventual changes in the modiolus porosity comparable to the modiolus samples shown in Fig. 4a. Nevertheless, correlations of hearing function with anatomical data for new bone growth in CI recipients do exist. First, based on word recognition scores and light-microscopic observation of temporal-bone sections, Kamakura and Nadol^56^ found that CI success was negatively correlated with the fractional volume of new bone within the scala tympani, but not correlated with the fractional volume of fibrous tissue; it was positively correlated with the total number of SGCs. Second, in an in-vivo study using ultra-high-spatial-resolution CT, Heutink et al.^16^ showed that long-term residual hearing loss was significantly larger in the 68% of CI recipients presenting new bone growth than otherwise (23 dB versus 9 dB at 2 kHz). Here, when calculating the porosity, we have considered the newly grown bone or fibrosis in ST as an extended layer of the modiolus, such as in S1 and S2. As described in “Methods”, increasing the α value in Eq. (2) will add additional bone mass to the modiolus, which quantitatively represents the new bone growth or ossification that impedes current flow due to the increase of low conductive bone mass. Here, the electric conductivity of the new bone is the same as that of the modiolus bone material.

Figure 4 examines how changes in modiolus porosity (Fig. 4a) might influence (maximum) transmembrane potentials (Fig. 4b). Figure 4b presents the V_max_ of the five SGNs using either mapped conductivity (MapC) or effective conductivity (EffC) for the nine modiolus samples. Before the onset of neural degeneration or new bone growth, the effective conductivity of the (healthy) modiolus for 20% porosity is 0.2734 S m^−1^. The average V_max_ due to MapC and EffC is 1.5 mV and 3.0 mV, respectively. Taking these values as a reference, we now interpret the meaning of V_max_ for the nine samples.

The average V_max_ of the SGNs for the S2 modiolus calculated with MapC and EffC is reduced to 1.2 mV and 2.25 mV, respectively, from the control values of 1.5 mV and 3.0 mV. Here, the porosity decreased from 20 to 8.5%, representing severe ossification or new bone-tissue growth. Due to severe occlusion of the pores with non-conducting, extremely ossified bone or fibrous tissue in the S1 modiolus with zero porosity, the average V_max_ is further reduced to 0.8 mV for both the MapC and EffC approaches. These results predict that, for a given excitation threshold, the likelihood of nerve excitation by the CI is negatively correlated with the growth of non-conducting material in the pores. These predictions are consistent with in-vitro^56^ and in-vivo^16^ studies finding that auditory performance is negatively correlated with the amount of new bone growth. Here, the MapC approach shows a moderate drop in the V_max_, suggesting that the bone growth may gradually affect the CI outcome. In contrast, the EffC approach is associated with a drastic drop in V_max_, indicating the profound effect of extreme growth of non-conducting material on the CI outcome. However, several experimental studies, for example, reviewed in^57^, opine a moderate effect of modiolus ossification on the stimulation efficiency of the CI.

For the S3 modiolus with 23.5% porosity, which is close to the assumed porosity of a healthy and normal modiolus (20%), the average V_max_ is 2.0 mV and 3.5 mV for MapC and EffC, respectively; that is, a 0.5 mV increase relative to V_max_ for the healthy modiolus. The 3.5% increment in the porosity is the result of 8.75% neural degeneration. Similarly, for the S4 modiolus with 33.1% porosity caused by 32.5% neural degeneration, the average V_max_ increases by approximately 1 mV for both MapC and EffC. On the other hand, for the S5 modiolus with 43.5% porosity due to 60% neural degeneration, the average V_max_ is 3.3 mV for MapC and 4.5 mV for EffC. This significant increase of V_max_ indicates that even mild-to-moderate neural degeneration could have an impact on AN stimulation. Indeed, the analysis predicts that this amount of neural degeneration and the ensuing increase of porosity actually increase neural excitability. In contrast, based on theoretical considerations, the partial demyelination of peripheral processes (Fig. 1a) increases the threshold for cathodic but not anodic stimulation^58^. Experimental evidence for neural degeneration has been provided by an increase of cathodic threshold relative to anodic threshold in CI patients^59,60^. However, evidence for a threshold increase was found in “only” about 78% of cases^60^. Apart from a contribution to the subject variance from the electrode-to-modiolar wall distance investigated in^60^, the present analysis shows that modiolar porosity might also contribute to the subject variance. It is also worth noting that, although neural excitability might increase with porosity as derived here, the scope of selective stimulation of SGNs for better pitch perception and word recognition would decrease due to current spread. Clearly, the increment in V_max_ alone cannot guarantee a better CI performance, as the generation and propagation of action potentials elicited by a CI also depend on the health of the AN and SGNs^21,61^.

The S6 modiolus with 56.3% porosity resulting from 81% neural degeneration represents an extreme degenerated state with very little neural tissue. The average V_max_ is 4.2 mV for MapC and 4.5 mV for EffC. In such pathology, current pathways in the modiolus become highly conductive, allowing the injected current to readily flow through the modiolus. Clearly, in such extreme cases, the number of healthy residual SGNs would determine the advantage of having a less resistive interface for better CI output.

The S7, S8, and S9 modioli represent the modiolus malformations discussed in^62^, where the bony modiolus is partially or completely absent. The average V_max_ is 4.44 mV, 4.63 mV, and 4.83 mV, respectively, for MapC. These values are similar for EffC, being 4.61 mV, 4.71 mV, and 4.73 mV, respectively. The V_max_ are very similar for these three cases due to the near-absence of a modiolus. This result shows that for extreme porosity caused by a modiolus malformation, CI performance may not depend on the electrical characteristics of the modiolus.

### Utility of the mapped and effective conductivity approaches for in-silico modelling

These two newly developed methods of mapped conductivity and effective conductivity provide a theoretical framework for examining the dependence of electric fields and potentials on the porosity of the modiolus under normal and pathological (neural degeneration and new bone growth) conditions. Indeed, the conductivity map, by definition, mirrors the spatial distribution of the pores. As shown in Fig. 4b, both methods resulted in approximately the same values of V_max_ for the S1, S7, S8, and S9 modioli which simulate extreme neural degeneration. In these cases, the effective conductivity of the modiolus is either too low or too high with respect to, for example, the accepted value of 0.2 S m^−1^, after optimizing 16 patient models described in^63^, or that measured in animal experiments^27,49^.

The MapC and EffC methods yield considerably different values of V_max_ for the S2–S6 modioli. In-silico studies considering a moderate neural degeneration, such as in^34,43,46,47^, involve modioli corresponding to S2–S6. If such models were to study the effects of modiolus porosity with the aid of the EffC method, the parametric sweep of electrical conductivities for the S2–S6 modioli would not show a significant change in the V_max_, even after translating the increased modiolus porosity to a higher conductivity value. Based on such study results, one would conclude that the impact of modiolus porosity on neural excitation is negligible. In contrast, with the aid of the MapC method, even a small amount of neural degeneration can become evident in the modiolus conductivity, thus uncovering a possible dependence of neural excitability on porosity.

The EffC method did not yield a significant change in V_max_ after the initial (approximately) exponential increase up to the S3 modiolus, implying that the EffC method would only weakly capture the electrical response of the modiolus in the event of neural degeneration. In other words, assigning a homogeneous electrical conductivity to the modiolus would not be a feasible approach for studying the effect of neural degeneration on the excitability of the AN. Also, simply removing the peripheral processes from the cochlear model as implemented in^34,43,64^ will not necessarily capture the effect of physiologically realistic neural degeneration nor the electrophysiological responses within the modiolus. Furthermore, tissue degeneration itself could alter the electric field distribution in RC^65^. Hence, by using the MapC method, a major modelling pitfall of neglecting a significant morphological change in the modiolus induced by cochlear pathology can be avoided.

### Effects of random pore distribution

Physiologically, if two CI recipients with similar initial pathology were subjected to a comparable rate of neural degeneration or new bone growth in a similar cochlear region, the overall porosity and the effective conductivity of both modioli samples, derived from the image-based model of the modiolus, would remain the same. Therefore, a similar neural output for both recipients would be expected. Consequently, parameter changes in the model would not be required to study those cases individually. However, their distribution of pores induced by pathology may not be identical. Therefore, we consider it critically important to study whether modiolus samples having the same porosity but different pore distributions might result in different neural sensitivities to CI stimulation.

Modiolus samples modelled using SEM images do not fit this particular scenario since each sample would have different overall porosity. Modiolus pores seem to form random patterns due to their varying size, shape or distribution. We have simulated these random patterns using coupled reaction–diffusion equations similar to those employed to mimic morphogenesis phenomena by Allen Turing and others^66^. We have employed two coupled reaction–diffusion equations, originally described by Barkley^67^, modified by Bär and Eiswirth^68^, and further adapted for the present study. The details are presented in the “Methods”. The proposed ad-hoc equation system produces a kinetic distribution of pores in different modiolus regions keeping the global porosity almost constant for all time steps. For convenience, we call these equations ‘regionally kinetic’ (RK) porosity equations.

**Figure 5:**
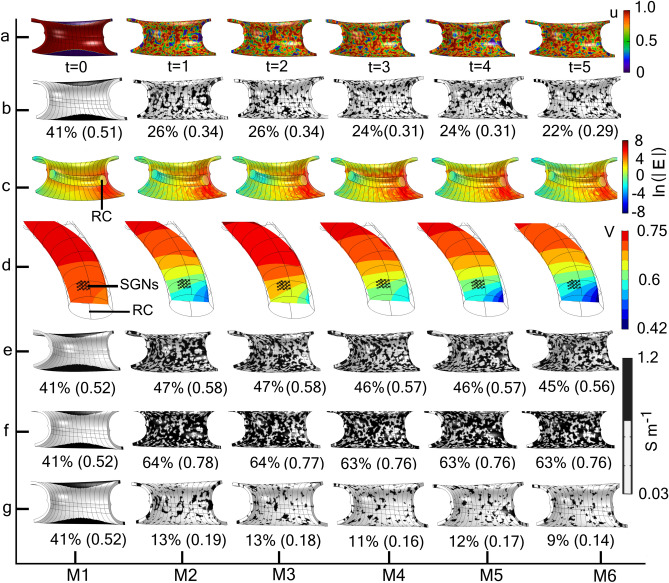
Generation of random distributions of modiolus porosity using regionally kinetic porosity equations and their associated electrical conditions. (**a**) Random pattern distribution of the state variable *u* defined by two coupled reaction–diffusion equations (“Methods”, Eq. (5)) for six time steps, generating six modiolus samples labelled M1–M6. For a given modiolus sample, the set of *u* values simulate the pixels of a colour image of the modiolus during the time stepping whilst solving the reaction–diffusion Eq. (5) (“Methods”). (**b**) Random distributions of electrically conducting pores (black regions) for M1–M6 based on the binary conditional Eq. (7) (“Methods”), with control parameter $\upgamma = 0.6$. Values in brackets are the effective conductivities (S m^−1^) calculated with Eq. (4) using the Wiener upper bound. Notice that the five samples M2–M6 present different pore patterns (black regions) but all have approximately the same overall porosity (25%) and effective conductivity (0.32 S m^−1^). (**c**) Electric field distributions for M1–M6 expressed in natural logarithms relative to 1 V m^−1^. All subdomains except Rosenthal’s canal (RC) are kept hidden for better visualization. (**d**) Electric potential distributions in RC for M1–M6. The matrix of SGNs is indicated in each RC. Random distributions of electrical conductivity for M1–M6 having porosity (for M2–M6) of approximately (**e**) 45%, (**f**) 64%, and (**g**) 12%, generated using the control parameter $\upgamma =0.4$, 0.2, and 0.8 respectively.

**Figure 6:**
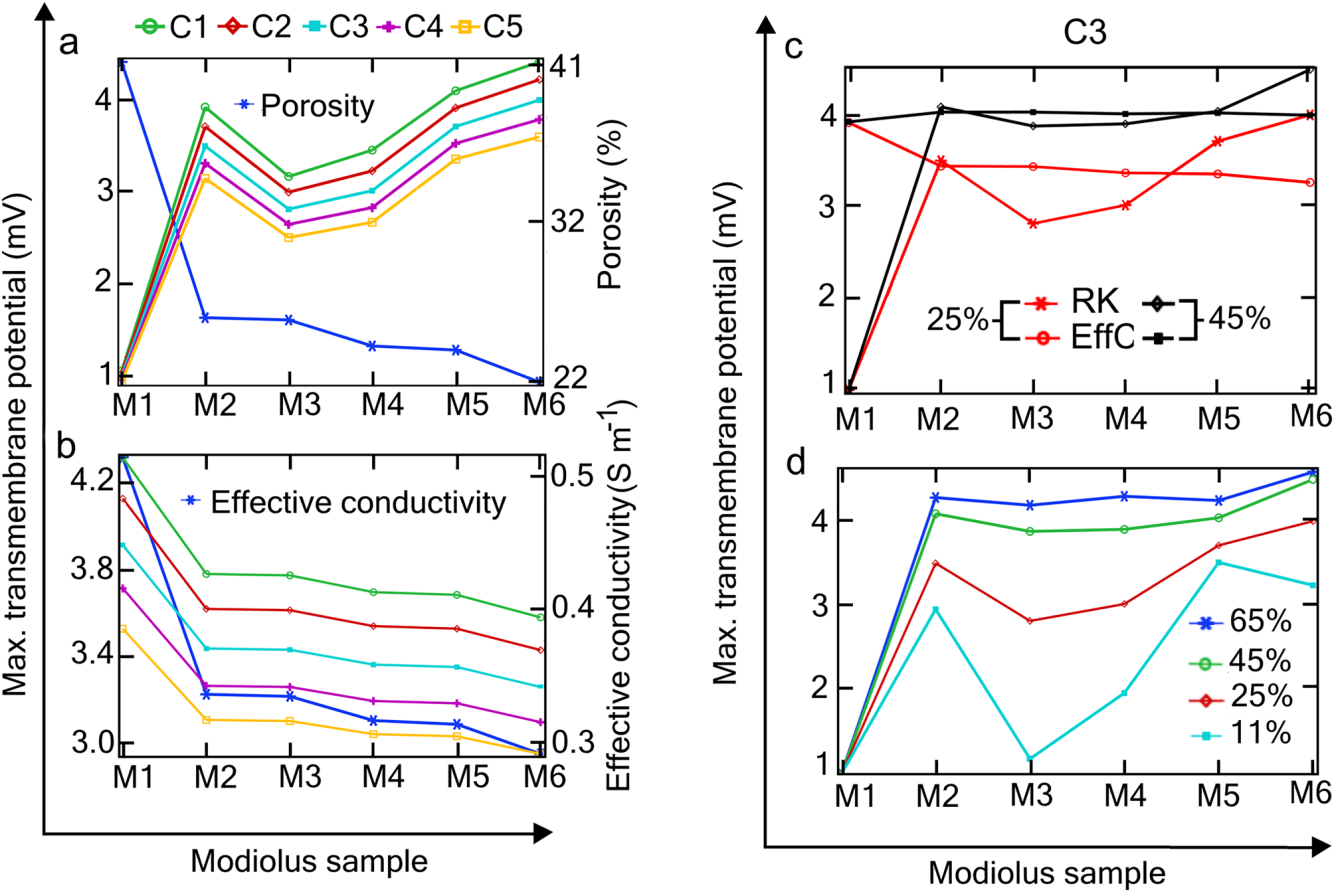
Dependence of the maximum transmembrane potential on porosity. (**a**) V_max_ of the five SGNs (C1–C5) for the six modiolus samples (M1–M6, Fig. 5b) based on the RK porosity equations. Notice that the two sample pairs M2 and M3, M4 and M5, have approximately the same overall porosity but significantly different V_max_ due to their different pore distributions. (**b**) V_max_ of the same SGNs and modioli but calculated with the effective conductivity rather than the mapped conductivity as in (**a**). In contrast to that achieved with mapped conductivity, the V_max_ are approximately the same for M2–M6. The ordinate in this panel is magnified relative to those in the other panels. (**c**) V_max_ for the SGN C3 with modioli of approximately 25% (Fig. 5b) and 45% (Fig. 5e) porosity, where V_max_ is calculated using the effective conductivity (EffC) method and the mapped conductivity using RK equations (RK). Notice that for M2–M6 and 45% porosity, the V_max_ are similar when derived from EffC or MapC. (**d**) V_max_ for the SGN C3 for the four porosity groups given in Fig. 5; V_max_ is based on mapped conductivity. The continuous lines joining the V_max_ values in the four panels do not represent a functional relationship among the modiolus samples; instead, they serve as a visual aid.

Figure 5a shows the random patterns formed by solving the RK porosity equations for six time steps on the modiolus subdomain, yielding a unique pattern of colour code at each time step; the six samples are designated as M1*–*M6. The generated colour-code samples are transformed into randomly distributed, electrically conductive pore distributions in the modiolus (Fig. 5b) by applying the binary conditional Eq. (7) (“Methods”), where the control parameter was set to $\gamma =0.6$. For each of M2–M6, this control parameter yielded approximately the same overall porosity (25%) and effective conductivity (0.32 S m^−1^). Whereas for M2–M6 the pores are randomly distributed, for M1 the pores are confined to two specific regions and yield higher overall porosity and effective conductivity. In spite of the similarity of porosity and effective conductivity for M2–M6, the distributions of the electric field intensities (Fig. 5c) and the voltage amplitudes (Fig. 5d) are significantly different. Consequently, the transmembrane potentials of the various SGNs also significantly differ dependent on with which modiolus the RC is associated, as illustrated in Fig. 6a for the five SGNs (C1–C5) located as given in Fig. 3a (inset). For example, a reduction of porosity of only 4% for M6 relative to M3 causes a 40% increase of V_max_ for the SGN labelled C1. The impact of pore distribution is even more evident for M1 where the porosity is approximately 42% yet V_max_ for the five SGNs is only about 1 mV, whereas for M6 it is 4.5–3.6 mV for C1–C5.

Figure 6c shows the effect of using effective conductivity rather than mapped conductivity as in Fig. 6a to calculate V_max_. Now, the V_max_ are approximately the same for M2–M6. Hence, the assumption of homogeneous effective conductivity in studies of neural degeneration would be inadequate.

The effect of porosity on V_max_ is illustrated in Fig. 6d for the four groups of porosity patterns presented in Fig. 4b,e–g. The porosities of 45% (Fig. 5e) and 64% (Fig. 5f) represent cases of profound neural degeneration. For these two porosity values, the randomness of the pore distributions has only a marginal effect on V_max_ for the modioli M2–M6 (Fig. 6d). The porosity of 12% (Fig. 5g) represents the case of increased ossification. Here, there is a profound effect of randomness on V_max_ for the modioli M2–M6, where an almost 300% variation in V_max_ is observed.

Taken together, these simulations suggest that under conditions of neural degeneration or modiolus ossification, the randomness of the distribution of pores would significantly affect neural excitability.

### Limitations and significance

To the best of our knowledge, there are no in-silico, in-vitro, or in-vivo studies with which we can compare or contrast the present results. The algorithms introduced here provide a novel theoretical framework for incorporating changes of modiolus porosity inflicted by neural degeneration or by osteopathology into in-silico studies and predicts electrical characteristics of the porous morphology pertinent to neural stimulation with a CI. As such, the results provide a framework for future hypothesis-based investigations of the dependence of CI outcome on the morphoelectrical properties of the modiolus.

Cochlear geometry derived from image stacks along the cochlea would have better captured the morphology. However, such an attempt demands exorbitant labour for image segmentation and prevents further case-specific, mathematical manipulations in the degree of porosity. Additionally, extremely fine mesh size and a large number of mesh elements are required to retain the shape of micro-pores in the modiolus. In contrast, the cochlear model developed in the present study is geometrically simple, computationally inexpensive, and easily adaptable to various study-specific modifications. For example, a patient-specific cochlear model could be built by extracting geometric coordinates using ultra-high-resolution computed tomography (CT)^16^ data to mathematically define the spiral structure of the cochlea and, in particular, of the modiolus.

The SEM image employed for the image-based, mapped electrical conductivity provides only a simplified impression of the distribution of modiolus pores. The linear interpolation of pixel intensities cannot accurately replicate the actual morphology of the porous modiolus. Nonetheless, we have persevered and used available SEM-image data for qualitative modelling to advocate the importance of considering the porous morphology of the modiolus to improve CI outcome and address the ‘enigma of poor performance’^12^.

The present simulation is confined to studying the effect of modiolus porosity on the induced transmembrane potential of SGNs and subsequent neural excitability by the CI. Clearly, several other morphological factors, such as tissue heterogeneity in RC, could equally affect CI outcome. Diverse cell-types such as Schwann cells, satellite glial cells and type-2 SGNs along with myelinated central axons in RC might profoundly affect the electric field distribution in RC^69^. Nevertheless, in spite of inherent computational limitations related to the enormity of the number of morphological variables in the real RC, we were able to partially model electrical properties of an inhomogeneous medium in RC employing a limited number of SGNs.

**Figure 7:**
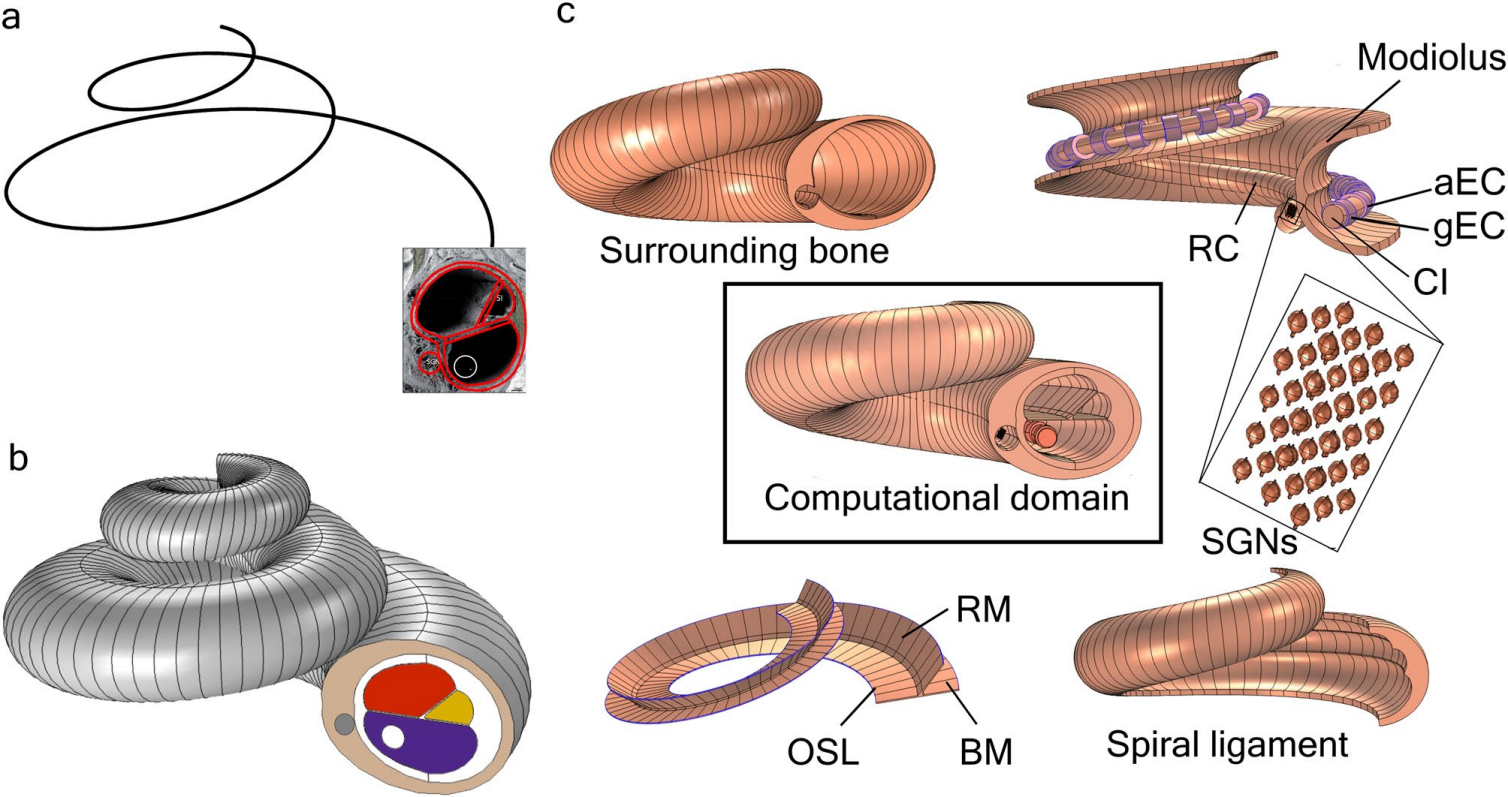
Three-dimensional morphological model of the cochlea with an inserted cochlear-implant electrode. (**a**) Logarithmic curve (black) defined by Eq. (1) and the subdomain surfaces (red contours) delineated from a cross-section of the human cochlea (Fig. 1b). (**b**) A full 3D model of the cochlea, red: scala vestibuli, yellow: scala media, blue: scala tympani, in which the cochlear-implant electrode (white dot) is located, green dot: Rosenthal’s canal. (**c**) Magnified view of the computational domain, showing the one-and-a-half turns of the cochlea implemented in this study and the orientation of 37 of the 45 spiral ganglion neurons (SGNs; zoomed inset). An SGN is composed of a spiral ganglion cell attached to both a peripheral and a central axonal initial segment. aEC: Active electrode contact, BM: Basilar membrane, CI: Cochlear-implant electrode, gEC: Ground electrode contact, OSL: Osseous spiral lamina, RM: Reissner’s membrane, RC: Rosenthal’s canal.

Although a variety of CI electrode designs are available on the market^70^, we arbitrarily chose a standard geometry for the CI electrode (Fig. 7c). Within the realms of the model, the current distribution in RC may alter with the design and placement of the CI electrode but should yield qualitatively similar simulation results. Nevertheless, the CI electrode model can be replaced with any desired design without altering any other subdomain in the model.

The present study depends heavily on conductive properties of cochlear tissues and fluids gleaned from multiple sources in the literature. Future efforts must experimentally acquire electrical material properties combined with morphological properties of the modiolus to enable reliable quantitative conclusions. Current developments of cochlear endomicroscopic systems by Stankovic and colleagues^71^ for use in humans both in vitro and in vivo, when combined with electrical sensors, promise a technological breakthrough that should allow morphoelectrical investigation of the modiolus.

We chose to simulate bipolar stimulation because of its inherently localised field properties. In the case of monopolar stimulation, to capture the field distribution between the intracochlear active electrode and the counter electrode necessitates a full-head computational model consisting of several different neural tissues with their associated electrical properties. Additionally, microanatomical structures, such as habenula perforata^72^ which may serve as a vital current pathway for monopolar stimulation, would need to be modelled precisely. This in turn would immensely increase the computational costs and the number of unreliable model parameters, such as dielectric properties of tissue, thus reducing the reliability of the simulation results.

The most significant advantage of the MapC method and RK porosity equations is the ease of their implementation in all established in-silico models. By incorporating these porosity equations into a morphoelectrical description of the modiolus, we expect—in general—that in-silico studies^23^ will become more relevant in a clinical setting with personalized medicine.

### Further applications

The identification of pathology-specific protein concentration in perilymph^73^, coupled with the investigation of resultant variations in the electrical conductivity of cochlear fluids, could serve as a prospective diagnostic and prognostic assay for hearing loss. The present modelling framework may serve as a first step for such investigations.

Furthermore, it would be interesting to investigate the influence of modiolus morphology on non-electric stimulation, such as optical stimulation^74–76^ of the auditory nerve, including infrared stimulation^74^ and optogenetics^75,76^. In such protocols, the optical response of tissue and bone may vary with the composition of the modiolus, which may reduce the spatial precision of neuronal stimulation. As with electrical stimulation, a dedicated experimental set up is required to examine the stimulation profile of the auditory nerve in optical or optogenetic fields under various conditions of modiolus porosity.

## Conclusion

Based on the results of the present study, we posit that the porous morphology of the modiolus is an essential factor determining the effectiveness of neural excitation with a CI. The desired random pore distribution of the modiolus can be implemented in any volume conductor model using the regionally kinetic porosity equations. The simulations show that if modiolar osteopathology and neural degeneration are translated in terms of an inhomogeneous distribution of modiolus conductivity, then the electrical characteristics of the modiolus could critically influence the CI outcome. Until now, the porosity of the modiolus is only discernible microscopically—its electrical properties remain latent. To unravel the impact of modiolus porosity on CI outcome, further in-silico investigations coupled with polarity sensitivity studies similar to Refs.^59,60^ are essential.

## Methods

### Modelling the 3D geometry of the cochlea

A ‘benchmark’ human cochlea for studying all modelling scenarios is not plausible due to the myriad of inter-individual anatomical variations of the cochlea^41,54^. As a compromise, depending on the requirements of a given study, cochlear anatomy can be often represented with a simplified volume-conductor model. In the present study, an intracochlear bipolar electrical stimulation configuration is used, where the active and ground electrodes are mutually adjacent in scala tympani of the basal turn, meaning that the current is localised in RC. Since the current follows the least resistive path to reach the ground electrode, the electrical properties of tissue interfaces between the electrode and the neural counterparts play a dominant role compared to those of the bony labyrinth far from the CI electrode. Therefore, in the present stimulation configuration, a simplified three-dimensional (3D) model of the cochlea should yield meaningful simulation results. Hence, we considered a parametric model of the cochlea similar to those used in Refs.^32,33,63^.

The 3D geometry of the cochlea was modelled using COMSOL Multiphysics®5.5 (Sweden) simulation software, referred to as COMSOL throughout the paper. First, to mimic the spiral structure of the cochlea, the following 3D logarithmic spiral was generated in Cartesian coordinates:

1 $$\left. {\begin{array}{*{20}l} {\text{x} = \text{exp}\left( {0.25{\uptheta}} \right){\text{cos}}\left( {2.25{\uptheta}} \right)} \\ {\text{y} = \text{exp}\left( {0.25{\uptheta}} \right){\text{sin}}\left( {2.25{\uptheta}} \right)} \\ {\text{z} = \text{exp}\left( {0.25{\uptheta}} \right)} \\ \end{array} } \right\}$$

where ${\text{x}}$, ${\text{y}}$, ${\text{z}}$ are the coordinates and $0\le\uptheta \le 2\uppi$. In the simulation, the CI extends 1.5 turns into the cochlea (Fig. 7c), meaning that ${{\uptheta}_{\text{max}} = 4{\uppi}/3 \equiv 240^{^\circ }}$. This extent corresponds to the usual maximum, active insertion length chosen by the surgeon to reduce the likelihood of tissue damage^5^.

In the next step, a surface model of all essential components of the cochlea (illustrated in Fig. 7b) was constructed using the contours obtained from the cochlea cross-section image shown in Fig. 1b. In the final step, all necessary surfaces of cochlear structures were extruded along the logarithmic curve shown in Fig. 7a. The resultant 3D geometry of the computational subdomains was scaled-down to within the range of realistic dimensions of the cochlea^77^, as shown in Fig. 7b. A magnified view of the final geometry of the computational domain is shown in Fig. 7c. It should be noted that each subdomain is modelled separately to facilitate the individual simulation of neural and osseous pathologies. For example, the porosity of the modiolus subdomain can be modified without affecting the other subdomains.

### Modelling the auditory neural tissues in Rosenthal’s canal

Modelling a realistic geometry of the AN fibres and embedding them according to their spatial distribution in a volume conductor model of the human cochlea is an extremely complex modelling task. Therefore, in-silico studies have used lumped element models of the AN^34,78^. Since the AN fibres were not present in those volume conductor models of the cochlea, those in-silico models could not capture the morphological features of the AN fibres as computational domains. Hence, the effect of a resultant heterogeneous, extracellular medium on the electric field distribution in the region of interest was seldom studied^69^.

For the present study, we embedded 45 uniformly arranged SGNs in a 3 × 3 × 5 matrix in RC in the basal (high-frequency) region of the cochlea. The distance between geometric centres of any two adjacent SGNs is 60 µm. This density of SGNs is smaller than the anatomically observed density for a healthy cochlea—typically, for segment II of the cochlear-duct length ranging from an angular depth of about 75°–240°^52^, which encodes frequencies of 1.2—8 kHz, there are about 900 SGNs per millimetre for a young adult with normal hearing thresholds^52,79^. The number of SGNs was chosen as a compromise between the computational cost and the simulation accuracy of a physiologically realistic model embedded with several hundred SGNs. In the real cochlea, the SGN count can indeed fall as low as 45 SGNs per millimetre in the case of severe neural degeneration^52,79^. Hence, the modelled 45 SGNs are supposed to be surviving any degree of neural degeneration. Although it is not known where the action potential is initiated^80^, various studies suggest that the SGN cell body or the initial axonal section emerging from the habenula perforata are the most probable signal initiation sites on the AN during CI stimulation^81–83^. For this reason, we have modelled the geometry of the bipolar SGN cell body (30 µm diameter for type I SGNs^84^) attached to both peripheral and central axonal initial segments of 4-µm length with 2-µm and 4-µm diameter, respectively^46^. To mimic neural depletion in the osseous spiral lamina, we neglected the geometry of the peripheral processes of the AN (Fig. 1a). Also, we ignored the central processes of the AN because the present study is not intended to model neural propagation centrally.

Rather than focussing on action-potential initiation or propagation, the present study emphasizes the importance of the porous morphology of the modiolus for CI efficacy. To this end, we consider the initial response of the SGNs to the applied electric field as an indicator of action-potential initiation, rather than neural activation mechanisms, described for example by the Hodgkin-Huxley (HH) single neuron model^85^. As discussed in^86,87^, the neural membrane responds linearly to the applied electric field until the transmembrane potential reaches approximately 80% of the excitation threshold potential. Also, the subthreshold response to one pulse can be used by the neuron to facilitate the generation of an action potential in response to the next pulse^88^. Therefore, the subthreshold transmembrane potential of a passive neural tissue can serve as an early indicator of the imminent initiation of an action potential^89^. Hence, instead of simulating the action-potential initiation and propagation in the AN using multi-compartment cable models such as those discussed in^34,78^, we chose to simulate the subthreshold transmembrane potential of the SGNs. The subthreshold potential values obtained in the present study could be readily implemented in a lumped element model of AN excitation based on the HH single neuron model. Nevertheless, due to the nonlinear nature of the HH model, such an attempt would severely complicate our investigation at the risk of not being able to understand possible effects of modiolus porosity on AN excitation. Although the excitation of SGNs depends on many factors^88,90–92^, variation in the maximum induced transmembrane potential could evince the likelihood of AN excitation.

### Modelling the modiolus porosity

State-of-the-art in-silico models of the cochlea (for a review see^93^) have neglected the porous morphology of the modiolus and simplified it as a homogeneous bone. Consequently, the models have assigned an isotropic electrical conductivity value to the modiolus for simulations of AN stimulation. Possible reasons for such simplification could firstly be that the porous morphology captured in microcomputed tomography (µCT) or magnetic resonance imaging (MRI) images have insufficient resolution to reconstruct the geometry of the porous modiolus by using image segmentation techniques. Secondly, except for scanning electron microscopy (SEM) images of the modiolus^7,9,10,48,94^, standard data regarding the shape, size, distribution, and volume fraction of the pores are not available. Thirdly, although the porous nature of the modiolus is evident from morphological studies, a suitable modelling method has not been available for in-silico studies. Finally, it has been tacitly assumed that the effect of modiolus porosity on AN stimulation would be negligible.

It can be observed from the images published in^7,9,10,48^ that the pores have neither a defined shape nor a prescribed spatial distribution along the modiolus. Hence, the geometry of heterogeneously distributed pores cannot be modelled in the modiolus subdomain. Nevertheless, in the present study, we took advantage of this peculiar randomness in pore sizes and distribution to capture the porosity of the modiolus in two scenarios. In the first scenario, we used an SEM image (Fig. 2a) of the porous modiolus to model nine samples of modiolus having different porosity. In the second scenario, instead of using image data for modelling porosity, we formulated an ad-hoc set of reaction–diffusion equations adapted from^66,67^ to form pore distribution patterns on the modiolus. Using the equation set, we modelled six samples of modiolus having similar overall porosity but different random distributions and pore sizes.

### Modelling porous modiolus morphology with SEM images

SEM images provide incredibly detailed visual data of a minuscule sample of the modiolus. Conceivably, in the framework of electrical stimulation of the AN by the CI, the electrical properties of the modiolus pores veritably contribute to the system. Therefore, we mapped the modiolus porosity in terms of the distribution of the electrical conductivity of pores on the bony modiolus. For convenience, we call such qualitative electrical mapping ‘mapped conductivity’. The proposed method is much simpler and largely different from that used for mapping conductivity tensors of human or animal brain using diffusion tensor MRI^95^.

We used the SEM image shown in Fig. 2a, depicting a part of the modiolus structure at the lower basal turn, to simulate the porosity of the modiolus in the computational model. We imported the pixel data of Fig. 2a into the COMSOL graphical interface, scaling the grey-scale intensity values between 0 and 1 and storing them as ${\text{Im}}({\text{x}},{\text{y}})$. Linearly interpolating the pixel intensities stored in ${\text{Im}}({\text{x}},{\text{y}})$, we mapped the isotropic conductivity of the bone-tissue complex (${\upsigma }_{{\text{b}}}$)—meaning bone together with neural tissue—and pores (${\upsigma }_{{\text{p}}}$) all along the 3D modiolus (${\upsigma }_{{\text{map}}}$) domain in accordance with the binary conditional equation:

2 $${\upsigma }_{{\text{map}}}=\left\{\begin{array}{*{20}l}{\upsigma }_{{\text{b}}}=0.0334\; (\text{S }{{\text{m}}}^{-1}),& \quad \text{Im(x,y)}>\upalpha \\ \\ {\upsigma }_{{\text{p}}}=1.2 \left(\text{S }{{\text{m}}}^{-1}\right). & \quad \text{Im(x,y)}\le 1-\upalpha \end{array}\right.$$

where $0\le {\upalpha }\le 1$ is the pixel intensity parameter. The value of ${\upalpha }$ controls the overall porosity of the modiolus; it will be used to generate modiolus samples of different overall porosity. Figure 2b shows the resultant inhomogeneous conductivity distribution on the modiolus for ${\upalpha }$ = 0.5.

The advantage of using the proposed image-based method to model the modiolus porosity is its ease of implementation and flexibility of modelling random variation in the porosity. Otherwise, it would be increasingly difficult and computationally expensive to handle the degree of variability in the mesh size while modelling the geometry of unstructured pores in a finite element model.

Alternatively, the conductivity of a composite material such as the porous modiolus can be characterized by its effective macroscopic electrical conductivity. Although several approaches are available as discussed in^96,97^, Wiener bounds^98^ provide the simplest analytical method for estimating the effective macroscopic conductivity of a multi-phase (two-phase) composite material. When the volume fractions and conductivities of the constituents are known, the maximum and minimum values of the effective electrical conductivity of the composite material can be estimated by the Wiener bounds. The Wiener upper and lower bounds are analogous to the upper and lower bounds of the equivalent conductivity of a network of resistors in parallel and series, respectively. It is apparent from the SEM image in Fig. 2a and the model modiolus shown in Fig. 2b that the pores are directed radially through the modiolus wall. Since modiolus pores filled with perilymph provide the least resistive path compared to that of the bone-tissue network, the applied current is expected to follow the direction of the pores. This expectation analytically yields the maximum attainable effective macroscopic conductivity of the porous modiolus.

After implementing the mapped conductivity on the modiolus subdomain, the volume fraction of pores (${{\text{V}}}_{{\text{p}}}$) and of the bone-tissue complex (${{\text{V}}}_{{\text{b}}}$) were estimated using:

3 $$\left.\begin{array}{c}{{\text{V}}}_{{\text{b}}}={{\text{V}}}_{{\text{Im}}\left({\text{x}},{\text{y}}\right)>{\upalpha }}/{{\text{V}}}_{{\text{mod}}}\\ \\ {\text{ V}}_{{\text{p}}}=1-{{\text{V}}}_{{\text{b}}}\qquad\qquad \end{array}\right\}$$

where ${{\text{V}}}_{{\text{Im}}\left({\text{x}},{\text{y}}\right)>{\upalpha }}$ is the volume occupied by the bone-tissue complex for a given intensity parameter ${\upalpha }$, ${{\text{V}}}_{{\text{mod}}}$ is the total volume of the modiolus subdomain; throughout, ${{\text{V}}}_{{\text{p}}}$ is called the overall porosity, normally quoted as a percentage.

Now that the conductivity and volume fraction of the pores and bone-tissue complex are known, the upper bound (${\upsigma }_{{\text{U}}}$) and the lower bound (${\upsigma }_{{\text{L}}}$) of the effective macroscopic conductivity of the porous modiolus (${\upsigma }_{{\text{eff}}}$) are estimated as:

4 $$\left.\begin{array}{c}{\upsigma }_{{\text{U}}}={\upsigma }_{{\text{p}}}{{\text{V}}}_{{\text{p}}}+{\upsigma }_{{\text{b}}}{{\text{V}}}_{{\text{b}}}\\ \\ {\upsigma }_{{\text{L}}}={\left[\frac{{{\text{V}}}_{{\text{p}}}}{{\upsigma }_{{\text{p}}}}+\frac{{{\text{V}}}_{{\text{b}}}}{{\upsigma }_{{\text{b}}}}\right]}^{-1}\end{array}\right\}$$

where ${\upsigma }_{{\text{L}}}\le {\upsigma }_{{\text{eff}}}\le {\upsigma }_{{\text{U}}}$; ${\upsigma }_{{\text{p}}}$ and ${\upsigma }_{{\text{b}}}$ are the conductivities of the fluid-filled pores and bone-tissue complex, respectively. As explained above, the orientation of the pores appears to be approximately parallel to the electric field, so that ${\upsigma }_{{\text{eff}}}$ will be approximated by ${\upsigma }_{{\text{U}}}$; that is, ${\upsigma }_{{\text{eff}}}\approx {\upsigma }_{{\text{U}}}$.

It should be noted that the mapped conductivity (${\upsigma }_{{\text{map}}})$ refers to the distribution of local conductivity based on the respective image intensity data, whereas the effective conductivity (${\upsigma }_{{\text{eff}}})$ refers to the global conductivity of the modiolous calculated using the volume fraction of constituents.

We did not model the peripheral processes of auditory tissues protruding through the modiolus. Instead, considering the highly porous nature of the modiolus, we have assumed that the functionally intact modiolus is composed of 40% bone, 40% myelin neural tissues, and 20% pores filled with perilymph (relevant data not available). Here, the conductivities of bone, myelin tissue, and perilymph are assumed to be 0.0836 S m^−1^^28^, 1.2 × 10^−6^ S m^−1^^40^ and 1.2 S m^−1^, respectively. Then, from Eq. (4), the (upper bound of the) effective conductivity of the healthy modiolus (before neural degeneration) would be 0.2734 S m^−1^. This estimate is within the range of conductivity values in previously cited experimental (e.g., 0.20‒0.23 S m^−1^ for the gerbil modiolus in the basal turn in vitro and in vivo^27,49^) and modelling (e.g., 0.33 S m^−1^ extracellularly in Rosenthal’s canal^99^) studies. The effective conductivity of only the bone-tissue complex (i.e., excluding the volume of pores filled with perilymph) would according to Eq. (4) be 0.0334 S m^−1^, the value used for modiolus bone in^34^. If, for example, neural degeneration results in 75% of neural tissue being replaced by perilymph, then the porosity would increase from 20 to 50% and the effective conductivity would increase to 0.6334 S m^−1^ (please refer to Supplementary material for details). An increase in modiolus conductivity was measured experimentally two months after neomycin-induced neural degeneration in the gerbil, from 0.23 to 0.35 S m^−1^^27^.

Electric conductivity values of all subdomains are given in Table 1.

**Table 1:** Electrical conductivity values of subdomains.

Subdomain	Conductivity (S m^−−1^)
Surrounding bone, excluding modiolus	0.016^34^
Electrode contact pads	9 × 10^6^ ^100^
Scala vestibuli	1.43^34^
Scala media	1.67^34^
Scala tympani	1.2*
Intracellular SGN fluid	0.3^101^
Intracochlear membranes (BM, RM, OSL, SL)	0.005^†^
Myelin tissue	1.2 × 10^–6^ ^40^

*Adapted for the present study (see *Introduction*). ^†^ Adapted for the present study from Refs.^31,100,102^. SGN: Spiral ganglion neuron; BM: Basilar membrane; RM: Reissner’s membrane; OSL: Osseous spiral lamina, SL: Spiral ligament.

### Modelling random pore distribution on the modiolus with reaction–diffusion equations

To simulate random patterns of pores in the modiolus, we used two coupled reaction–diffusion equations, originally described by Barkley^67^, modified by Bär and Eiswirth^68^, and further adapted for the present study. A comprehensive discussion of the system properties is outside the scope of the present report. It suffices to say that the proposed ad-hoc equation system produces a kinetic distribution of pores in different modiolus regions keeping the global porosity almost constant for all time steps. For convenience, we call these equations ‘regionally kinetic’ (RK) porosity equations.

The reaction–diffusion equations used here are:

5 $$\left.\begin{array}{c}\begin{array}{c}\frac{\partial {\text{u}}}{\partial {\text{t}}}=\frac{1}{{\text{c}}}\left({\text{u}}\left(1-{\text{u}}\right)\left({\text{u}}-\frac{\left({\text{v}}+{\text{b}}\right)}{{\text{a}}}\right)\right)+\text{D}\Delta \text{u}\\ \\ \frac{\partial {\text{v}}}{\partial {\text{t}}}=\text{g}\left({\text{u}}\right)-\text{v}-\upbeta \text{u} \;\qquad\qquad\qquad\qquad\end{array}\\ \end{array}\right\}$$

where ${\text{g}}({\text{u}})$ is defined as:

6 $${\text{g}}\left({\text{u}}\right)=\left\{\begin{array}{*{20}l}0, & \quad \text{u}<\frac{1}{3} \\ 1-9\text{u}{\left({\text{u}}-1\right)}^{2}, & \quad \frac{1}{3}\le \text{u}\le 1\\ 1. & \quad \,\text{u}>1\end{array}\right.$$

${\text{u}}$ and ${\text{v}}$ are the state variables; ${\text{a}}$, ${\text{b}}$, and ${\text{c}}$ are the system parameters; $\Delta$ and ${\text{D}}$ are the Laplace operator and the diffusion coefficient of the ${\text{u}}$ state variable, respectively; β determines the amount of space occupied by the maximum value of u in the random patterns.

The advantage of modelling modiolus porosity using the system of RK porosity equations is the ease of modelling the heterogeneous distribution of pores in three spatial directions by controlling the value of the diffusion coefficient ${\text{D}}$. Also n-number of porous modiolus samples can be obtained by running n-number of time steps. For the simulations presented here, we assigned $\upbeta =0.1$, ${\text{D}}=1$, ${\text{a}}=0.64$, ${\text{b}}=0.02$, and ${\text{c}}=0.08$. In the Supplementary material, we examine the system properties of the equations proposed by Bär and Eiswirth^68^ and thereby explain the motivation for our choice of parameters.

To implement the RK porosity equations on the modiolus subdomain, we considered a shorter segment (a quarter turn rather than the one-and-a-half turns of the CI) of the cochlea to minimize the effect of modiolus curvature on the electrical responses to current injection. We solved the RK porosity equations on the modiolus subdomain in the finite-element volume conductor model of the cochlea with the time step (∆t) proportional to the element size (h) such that ∆t = h/10 as in^68^. The mesh element size on the modiolus subdomain was 1 µm; hence, we used a 0.1-µs time step to achieve the spiral breakup and subsequent random pattern formation. First, we solved the RK porosity equations for sixtime-steps to generate six different pore distribution patterns for the modiolus samples. Then, using the distribution of the diffusing state variable u of the RK porosity equation system, we mapped the electrical conductivity values of the bony network (${\upsigma }_{{\text{b}}}$) and the pores (${\upsigma }_{{\text{p}}}$) using the binary conditional equation:

7 $${\upsigma }_{{\text{map}}}=\left\{\begin{array}{*{20}l}{\upsigma }_{{\text{b}}}=0.0334 \left(\text{S }{{\text{m}}}^{-1}\right), & \quad \text{u}>\gamma \\ \\ \;\quad{\upsigma }_{{\text{p}}}=1.2 \left(\text{S }{{\text{m}}}^{-1}\right). & \quad \text{u}\le 1-\gamma \end{array}\right.$$

where the control parameter $0\le\upgamma \le 1$ was taken between 0.8 and 0.2 to vary the modiolus porosity (${\upsigma }_{{\text{map}}}$) between 10 and 65%, respectively. The value of $\upgamma$ controls the overall porosity of the modiolus as ${\upalpha }$ does for the SEM imaged-based modiolus model. Figure 5a shows a map of ${\text{u}}$ for six time instants, generating six different modioli, and Fig. 5b displays the resulting conductivity distributions, ${\upsigma }_{{\text{map}}}$, for the six modioli using $\upgamma$ = 0.6.

### Simulation of electric fields and voltages

We simulated the electric field distribution in a volume conductor model of the cochlea, assuming all cochlear tissues are purely resistive within the quasi-static regime^103,104^. This assumption is supported by experimental data in the gerbil where it was found that the reactive component in the impedance measurements at the modiolar wall is negligible (phase changes < 10°) between 0.1 and 10 kHz^27^. The following set of equations was solved in the AC/DC module of COMSOL:

8 $$\left.\begin{array}{c}\bf{J}=\sigma \bf{E} \\ \quad \bf{E}=-\nabla {\mathrm{V}}\\ \nabla \cdot (\nabla {\mathrm{V}})=0\qquad\qquad\end{array}\right\}$$

where, $\mathbf{J}$, $\mathbf{E}$, ${\text{V}}$, and $\upsigma$ denote the current density, electric field, electric potential, and conductivity of the media, respectively. The solution of Eq. (8) was found for the following boundary conditions:

- The electric insulation boundary condition was assigned on all outer boundaries of the computational domain:
  9 $$\mathbf{n}\cdot \mathbf{J}=0$$
  where $\mathbf{n}$ is the unit normal vector and $\mathbf{J}$ is the current density.
- The continuity boundary condition was assigned to all interior boundaries except on the boundaries of SGNs and axonal initial segments:
  10 $$\mathbf{n}\cdot \left({\mathbf{J}}_{1}-{\mathbf{J}}_{2}\right)=0$$
  where ${\mathbf{J}}_{1}$ and ${\mathbf{J}}_{2}$ are the current densities on either side of the selected boundaries.
- For bipolar stimulation, using the Dirichlet boundary condition, a static 1V voltage was applied to one electrode contact and the adjacent electrode contact was grounded. All other electrode contacts were open-circuited. For the data presented in Figs. 3, 4, 5 and 6, the basal-most electrode contact was the ground and the current was applied to the next electrode contact.
- To model electrical conditions at the thin membrane (thickness, ${\text{d}}$ = 10 nm) of the SGC, the following contact impedance boundary condition was assigned to the boundaries of SGNs:
  11 $$\left.\begin{array}{c}\bf{n}\cdot {\mathbf{J}}_{{\text{int}}}=\sigma ({{\text{V}}}_{{\text{int}}}-{{\text{V}}}_{{\text{ext}}})/\text{d} \\ \bf{n}\cdot {\mathbf{J}}_{{\text{ext}}}=\sigma ({{\text{V}}}_{{\text{ext}}}-{{\text{V}}}_{{\text{int}}})/\text{d}\\ {{\text{V}}}_{{\text{m}}}={{\text{V}}}_{{\text{int}}}-{{\text{V}}}_{{\text{ext}}}\;\;\;\end{array}\right\}$$
  where ${{\text{V}}}_{{\text{m}}}$, ${{\text{V}}}_{{\text{int}}}$, ${{\text{V}}}_{{\text{ext}}}$, ${\mathbf{J}}_{{\text{int}}}$, and ${\mathbf{J}}_{{\text{ext}}}$ are the transmembrane potential, intracellular potential, extracellular potential, intracellular current density, and extracellular current density, respectively.

Thin and irregular subdomains in the computational domain were meshed manually, which resulted in 10,725,528 tetrahedral elements. The stationary solver and the time-dependent solver to solve the quasi-static problem and the RK porosity equation system, respectively, were automatically chosen by COMSOL. The time taken to solve the quasi-static problem was two hours, and to solve the RK porosity equation system was seven hours, on a Windows server workstation with 64-Bit Intel(R) CPU with 3.40 GHz (two processors) with 256 GB RAM.

### Supplementary Information

Supplementary Information.

## Data Availability

The modelling process presented in the “Methods” section is self-explanatory. However, if needed, a step-by-step tutorial on cochlea models used in the present study will be made available on GitHub upon request to the corresponding author: kiran.sriperumbudur@uni-rostock.de (alternatively, kiran.sriperumbudur@medel.com), or ursula.van-rienen@uni-rostock.de.
