# Supplementary material for: Understanding the impact of modiolus porosity on stimulation of spiral ganglion neurons by cochlear implants
Source: Sci Rep. 2024 Apr 26;14:9593. doi: 10.1038/s41598-024-59347-2 (PMC11053021; doi:10.1038/s41598-024-59347-2)
Supplement: Supplementary file 1 — Supplementary Information. [file 41598_2024_59347_MOESM1_ESM.pdf]

Supplementary material

A. Mesh selection for the 3D computational domain of the cochlea.

In the present study, the 3D spiral computational domain of the cochlea, with a few millimeters in length, contains thin membrane structures and as small as 2-μm axonal initial segments attached to the spiral ganglion neuron (SGN) cell body of 30-μm diameter. For the finite-element discretization, these three orders of magnitude in variations of the model geometry demand a large number of mesh elements to restore the shape of the extremely small subdomains. COMSOL Multiphysics® (briefly called COMSOL hereafter) finite-element software offers automatic inbuilt meshing options suitable for the computational domain according to the chosen study module; in the present case, the AC/DC module.

We studied all seven automatic meshing options and related mesh parameters *viz.* maximum element size (Element size) that limits how large each element could be, maximum element growth rate (Growth rate) that limits the size difference of two adjacent elements, curvature factor (Curvature factor) that limits how large a mesh element can be along a curved boundary, and resolution of narrow regions (Resolution of regions) that controls the number of layers of mesh elements in a narrow region chosen by COMSOL. Supplementary Table 1 shows the above-mentioned mesh parameters, solution time, and degrees of freedom (DoF) solved for each mesh option. Taking the numerical solution (transmembrane potential of an SGN) obtained by the finest mesh (mesh number 7) as the best solution ($\emptyset_{best}$), the relative (ϵ_{rel}) and the absolute (ϵ_{abs}) error magnitudes were calculated, respectively, according to equations (1) and (2), using numerical solutions obtained with the remaining meshes ($\emptyset_i$). As an eighth mesh, a manual meshing option was chosen to reduce the number of mesh elements while taking care of the thinnest subdomains, the computational cost, and the accuracy of the solution.

The dependence of ϵ_{rel} and ϵ_{abs} on the mesh type is shown in Supplementary Fig. 1a. Since the heterogeneous electric conductivity distribution was modeled on the modiolus subdomain, an adaptive mesh refinement was implemented on the modiolus to minimize the interpolation of conductivity values. Supplementary Figure 1b shows the final tetrahedral mesh obtained by the manual meshing (mesh number 8) used for all simulations in the present study.

$$\epsilon_{abs} = \|\emptyset_{best} - \emptyset_i\|_1 \tag{1}$$

$$\epsilon_{rel} = \frac{\|\emptyset_{best} - \emptyset_i\|_1}{\|\emptyset_{best}\|_1} \tag{2}$$

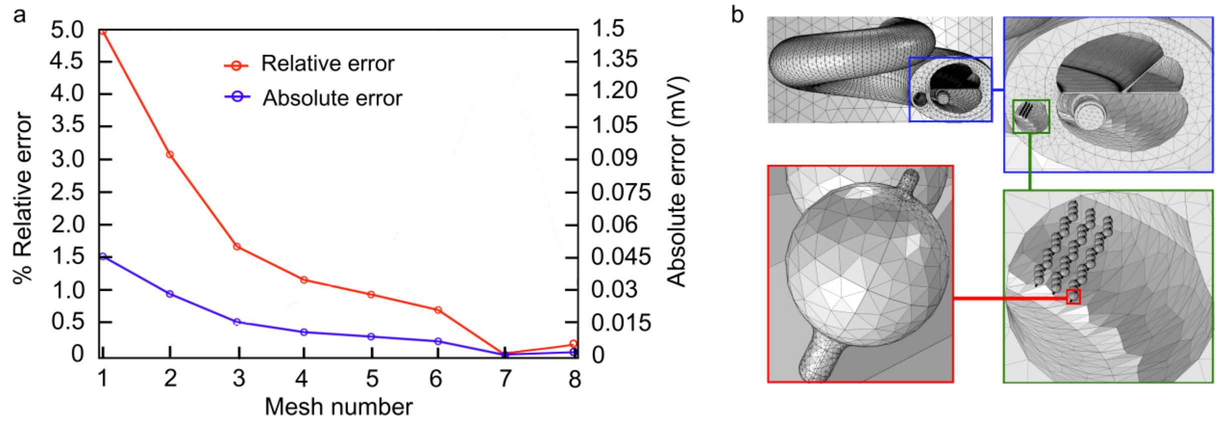

Supplementary Figure 1 | Finite-element mesh selection for the 3D computational domain. a, Relative error percentage and absolute error magnitude in numerical solutions while using automatic meshes generated by COMSOL (mesh numbers 1–7) and a manual mesh (mesh number 8). **b**, Finite-element mesh (mesh number 8) generated by manual meshing on the 3D computational domain. Several thin subdomains and their respective meshes are shown in the zoomed pictures.

Supplementary Table 1: Mesh parameters

Mesh. Number	Mesh type	Element size (mm)	Growth factor	Curvature factor	Resolution of regions	DoF	Solution time (s)
1	Extra coarse	0.47	1.7	0.8	0.3	1,562,443	464
2	Medium coarse	0.37	1.6	0.7	0.4	2,476,013	626
3	Coarse	0.24	1.5	0.6	0.5	3,850,053	1,092
4	Normal	0.19	1.45	0.5	0.6	6,190,217	2,423
5	Fine	0.13	1.4	0.4	0.7	9,401,537	4,095
6	Medium fine	0.08	1.35	0.3	0.85	15,993,600	6,140
7	Extra fine	0.04	1.3	0.2	1.0	30,501,300	14,611
8	Manual	0.05	1.45	0.5	0.6	13,618,181	4,200

B. Parametric study of 'regionally kinetic' porosity equations on a two-dimensional domain.

The present study proposes equations (3) and (4) to model the random pore distribution on the modiolus. This system of reaction-diffusion equations forms regionally kinetic (RK) random patterns for each time step after the spiral breakup while keeping the area occupied by the higher values of the state variable u almost constant in the computational domain.

For β = 0, k = 6.75, and D = 1, equations (3) and (4) become the system of reaction-diffusion equations proposed by Bär & Eiswirth [1], who have conducted several numerical experiments by choosing different values for the system parameters a, b, and c. They modified the piecewise linearized FitzHugh-Nagumo equations proposed by Barkley [2] to study spiral-breakup and turbulence patterns in an excitable media. Bär & Eiswirth studied the reaction-diffusion equation system proposed in [1] in a two-dimensional computational domain to reduce the computational cost while providing essential insights into the system dynamics. The parameter values tested through such studies can be used for the three-dimensional domain.

$$\left.\begin{aligned} \frac{\partial u}{\partial t} &= \frac{1}{c}\left(u(1-u)\left(u-\frac{(v+b)}{a}\right)\right) + D\Delta u \\ \frac{\partial v}{\partial t} &= g(u) - v - \beta u \end{aligned}\right\} \tag{3}$$

$$g(u) = \begin{cases} 0, & u < \frac{1}{3} \\ 1 - ku(u-1)^2, & \frac{1}{3} \le u \le 1 \\ 1. & u > 1 \end{cases} \tag{4}$$

In the present study, we assigned a = 0.64, b = 0.02, c = 0.08, D = 1, and β = 0.1. Here, we demonstrate how the newly introduced term −βu and the value k = 9 affect the system dynamics compared with the Bär and Eiswirth (BE) model and how the parameter β impacts the dynamics of the RK porosity equations on a square domain of length L = 3 mm and equidistant grid size h = 50 μm. The time step is similar to that in Barkley's model [2]; i.e., proportional to the square of the grid size such that $\Delta t = 0.4h^2 = 1$ ns. Studying the BE and RK equation systems by choosing such a time step facilitates a better understanding of critical transition points, such as spiral wave formation, wave breakup, and random pattern formation. The number of solved DoF was 30,000 using a time-dependent solver; the solution time for both BE and RK equations was 2.5 minutes to complete 500-time steps.

Supplementary Figures 2a and b show the progress of the spiral wavefront while solving the BE model after the indicated number of time steps (t). The spiral wavefront formation continued for the first ten time steps, followed by a mild spiral breakup from the 25th time step, and entered into total chaotic turbulence after the 100th time step. Unlike the BE model, the RK equation system did not form spiral wavefronts at any instance during the solution. After forming a sort of "linear" pattern until the 25th time step, the RK model resulted in random pattern formation at the 50th time step (Supplementary Figure 2c).

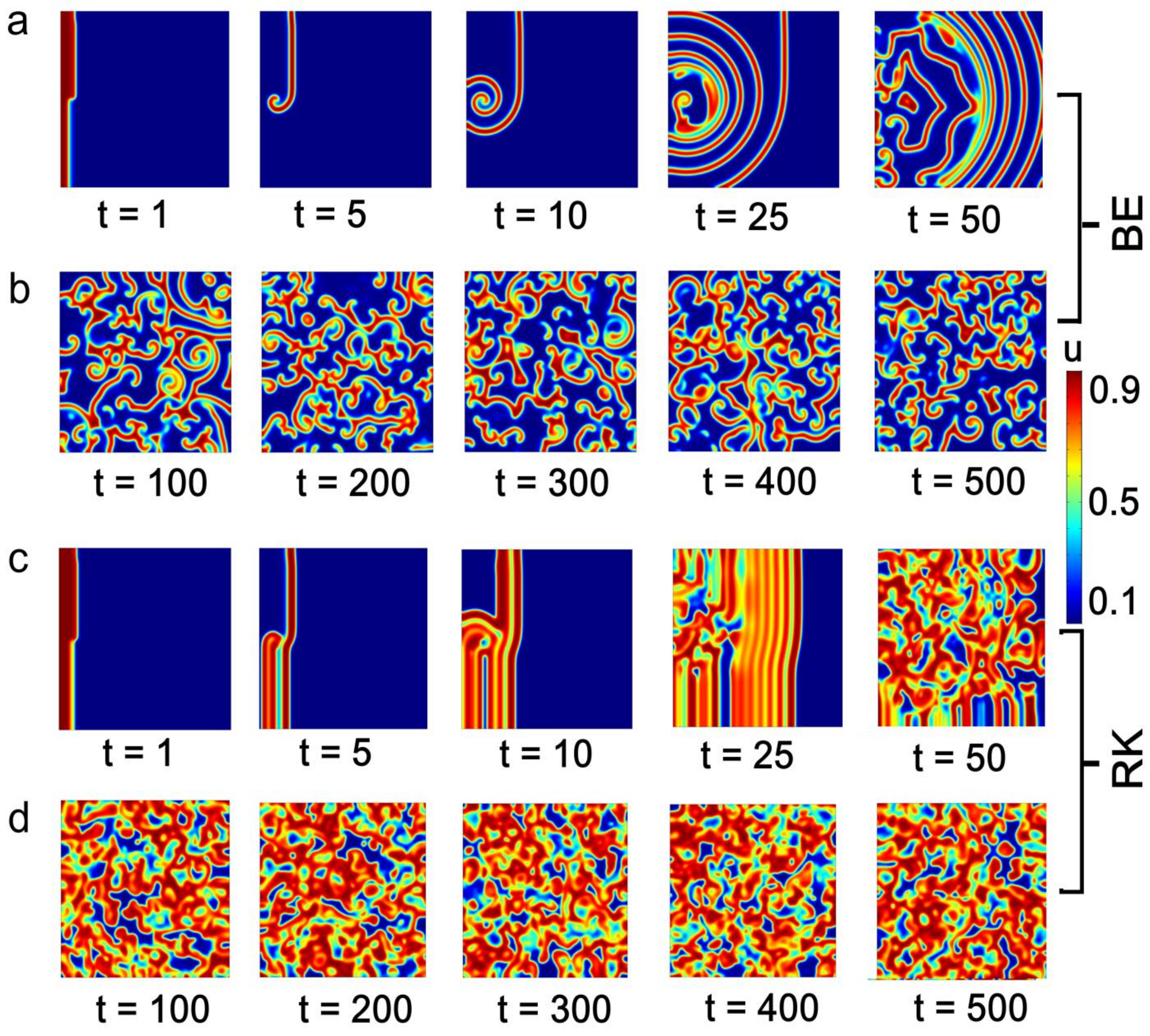

Supplementary Figure 2 | Numerical solution of the Bär & Eiswirth (BE) and regionally kinetic (RK) equations on a square domain of 3-mm length and 50-µm grid spacing. a & **b**, Solution of the BE model, and **c** & **d**, solution of RK equations, at the indicated number of times steps (t) for a time step of 1 ns. System parameters, a = 0.64, b = 0.02, c = 0.08, D = 1, and β = 0.1.

In the present study, the instants of interest are those generating random patterns by the RK equations from about the 100th time step (0.1 μs) onwards. Supplementary Figure 2d shows the formation of random patterns for β = 0.1, which are regionally kinetic and result in almost the same area occupied by the higher values of u. Supplementary Figure 3a shows the percentage of area occupied for each indicated value of u at each time step. Supplementary Figure 3b shows the impact of β on the spread of higher values of u and subsequent increment in its occupying area in the computational domain at the 100th time step. The β values higher than 0.2 resulted in an un-wanted spread of higher values of u (Supplementary Figure 3c). Hence, β should be chosen between 0.1 and 0.2, inclusive, for the RK equation system in the present application. Based on insights acquired from this comparative study, we assigned a = 0.64, b = 0.02, c = 0.08, D = 1, and β = 0.1 to solve the RK equations with the time step of 0.1 μs on the 3D computational domain of the modiolus.

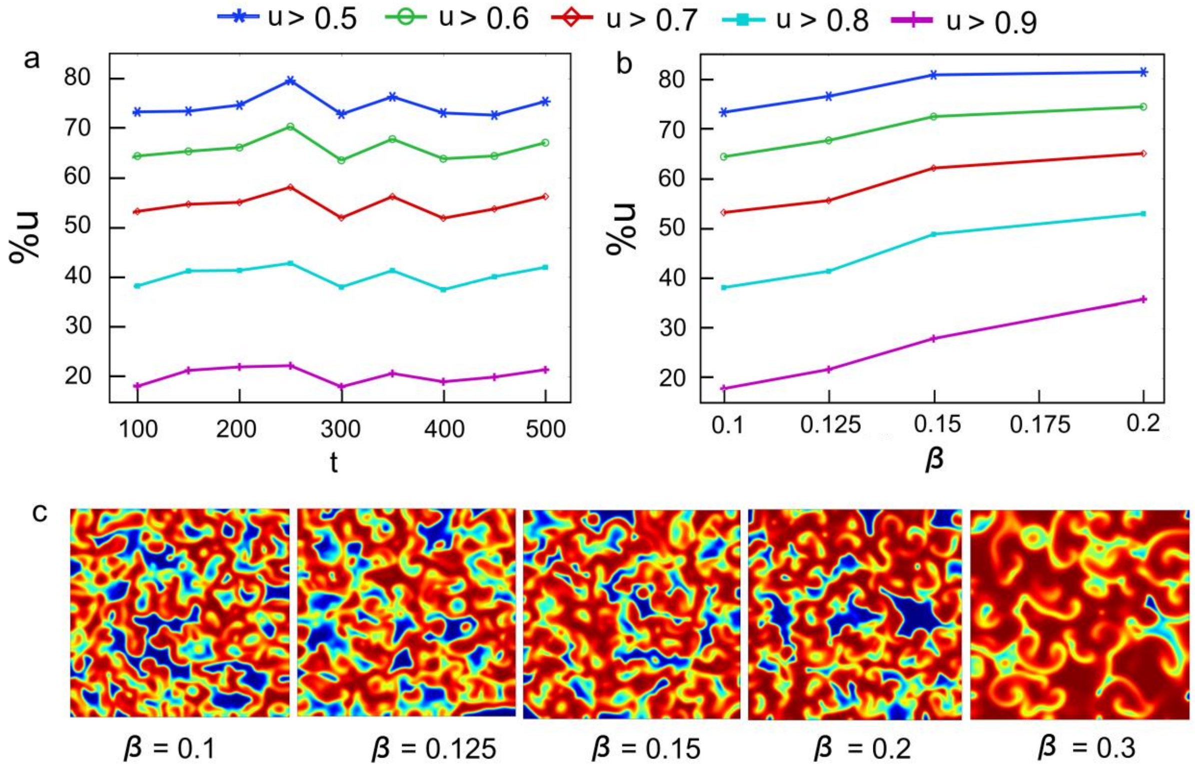

Supplementary Figure 3 | System properties of RK equations. a, Almost constant percentage of area (% u) occupied by the indicated higher values of the state parameter u at each time step (t) given in Supplementary Figure 2d. **b** & **c**, For t = 0.1 μs (100th time step), with small increments of β between 0.1 and 0.2, the higher values of u occupied more area in the computational domain. β = 0.3 resulted in an unwanted spread of u values.

C. Quantification of change in porosity induced by neural degeneration

We have assumed the modiolus is composed of 40% neural tissue, 40% bone, and 20% pores occupying the overall volume fraction of 0.4, 0.4, and 0.2, respectively. After, for example, 75% of neural tissue degeneration (75% of 0.4 is 0.3), only 0.1 volume fraction of neural tissue remains, and the empty space created by degenerated tissues is assumed to be occupied by perilymph. Therefore, 0.3 volume fraction is added to the volume fraction of the pores. Now, the new volume fractions of the neural tissue, bone, and pores is 0.1, 0.4, 0.5 (10%, 40%, and 50%) respectively. Hence, 75% neural degeneration would result in an increase of porosity to 50% from 20%.

References

1. Bär, M. & Eiswirth, M. Turbulence due to spiral breakup in a continuous excitable medium. *Phys. Rev. E* **48**, R1635–R1638 (1993).

2. Barkley, D. A model for fast computer simulation of waves in excitable media. *Phys. D Nonlinear Phenom.* **49**, 61–70 (1991).
